# Recyclable Cu(i)/melanin dots for cycloaddition, bioconjugation and cell labelling†
†Electronic supplementary information (ESI) available. See DOI: 10.1039/c6sc01536k


**DOI:** 10.1039/c6sc01536k

**Published:** 2016-05-20

**Authors:** Yao Sun, Suhyun Hong, Xiaowei Ma, Kai Cheng, Jing Wang, Zhe Zhang, Meng Yang, Yuxin Jiang, Xuechuan Hong, Zhen Cheng

**Affiliations:** a Molecular Imaging Program at Stanford (MIPS) , Bio-X Program , Department of Radiology , Canary Center at Stanford for Cancer Early Detection , Stanford University , California 94305-5344 , USA . Email: zcheng@stanford.edu; b State Key Laboratory of Virology , Key Laboratory of Combinatorial Biosynthesis and Drug Discovery (MOE) , Hubei Provincial Key Laboratory of Developmentally Originated Disease , Wuhan University School of Pharmaceutical Sciences , Wuhan 430071 , China . Email: xhy78@whu.edu.cn; c Chinese Academy of Medical Science , Peking Union Medical College Hospital , Department of Ultrasound , Beijing , 100730 , China

## Abstract

Development of biocompatible and high-performance heterogeneous catalysts for bioconjugation and cell labeling is highly challenging. Melanin has previously been used as a target for melanoma imaging and therapy. Herein, this important biomarker was transferred into a novel catalytic platform. A biocompatible Cu(i)/melanin dot-based catalyst [Cu(i)/M-dots] was easily prepared and exhibited high catalytic activity and excellent reusability in various Cu(i)-catalyzed azide-alkyne cycloadditions (CuAAC). Furthermore, DNA bioconjugation was carried out efficiently using Cu(i)/M-dots under ligand-free and reductant-free conditions, and the Cu(i)/M-dots could easily be removed by centrifugation. Lastly, the integrin receptor (alkyne RGD targeted) of U87MG cells was effectively labelled with a fluorescent dye (Cyanine5.5 azide) in combination with Cu(i)/M-dots. These attractive properties of Cu(i)/M-dots render it a promising catalytic platform in bioconjugation and chemical biology applications.

## Abstract 2

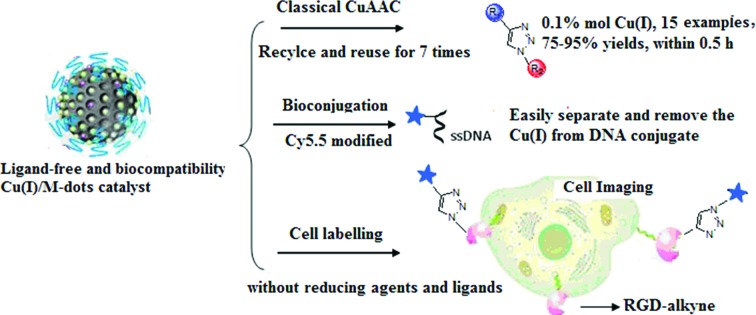
We successfully transferred melanin into a novel catalytic platform. Ligand-free, water-soluble, recyclable and biocompatible Cu(i)-loaded melanin dots [Cu(i)/M-dots] was easily prepared and demonstrate excellent properties for classic CuAAC, bioconjugation and cell labelling.

## Introduction

In recent years, tremendous efforts have been made in the development of biocompatible, biodegradable and renewable materials for various chemical and biomedical applications.^1^–^4^ Therefore, a variety of natural materials such as proteins, RNA, polysaccharides, *etc.*, have been actively explored to serve as molecular platforms to create next generation materials for drug delivery, imaging agents and theranostic applications.^5^–^7^ Melanin, a well-known biopolymer that is widely distributed in many living organisms, is particularly attractive owing to its excellent physical and chemical properties, including strong metal ion (Fe^3+^, Gd^3+^, Cu^2+^, Cu^+^, Zn^2+^, *etc.*) trapping ability and free radical quenching ability.^8^,^9^ Melanin has traditionally been used as a tumor biomarker for melanoma imaging and therapy.^10^ Recently, by mimicking natural melanin, our group has prepared artificial ultrasmall size (<10 nm) melanin nanoparticles (named as melanin dots, M-dots) and further used it as a nanoplatform for tumor multimodality imaging and drug delivery.^11^,^12^ It has been demonstrated that the M-dots platform not only exhibits good biocompatibility and water solubility, but also actively chelates various metal ions *via* its intrinsic chelating functional groups. Encouraged by these promising properties, we propose to use M-dots directly as a support for catalytic applications, which presents the following advantages: (1) the natural nitrogen groups of M-dots can coordinate metal ions and the resulting heterogeneous catalyst can greatly accelerate the kinetics of metal-catalyzed reactions; (2) the water-solubility of M-dots conveniently performs catalytic reactions under aqueous solvents and it can easily be removed by centrifugation; (3) the high stability and biocompatibility of M-dots are attractive for biological applications; (4) M-dots are able to scavenge radical molecules produced by metals under reducing conditions, which is one major challenges of current metal-mediated bioconjugate chemistry.^13^ Therefore, these excellent advantages could greatly expand the applications of heterogeneous catalytic systems in bioconjugation and chemical biology.

Within the last decade, bioorthogonal reactions have become a powerful tool for the selective modification of biomolecules in living systems.^14^,^15^ One hallmark of bioorthogonal chemistry is the Cu(i)-catalyzed azide-alkyne cycloaddition (CuAAC) click reaction which was discovered by Sharpless and Meldal in early 2000s.^16^,^17^ However, the use of the CuAAC in a bioconjugation and cellular context has been limited because of the toxicity associated with Cu(i).^18^ For example, recent studies have shown that the Cu(i)-mediated generation of reactive oxygen species (ROS) from O_2_ can cause detrimental consequences to cellular metabolism.^19^ To address this problem, several water-soluble Cu(i) ligands have been designed as Cu(i) stabilizers to reduce the cellular toxicity of copper.^20^ Another approach was recently reported by the Ting and Taran groups, using highly reactive azides according to the concept of a chelation-assisted CuAAC reaction.^21^,^22^ Despite the significant progress made, it is still very difficult to remove residual Cu(i) from conjugated products. This raises concerns about protein or DNA damage/precipitation and the preservation of their structural integrity.^13^ Furthermore, in order to stabilize the Cu(i) oxidation state for efficient catalysis, a large amount of chelating ligands and reducing agents are usually required. It could induce further damage to cellular metabolism during a labeling reaction.^23^ These challenges prompted us to explore a novel recyclable Cu(i) catalyst that performs click reactions under ligand-free and reductant-free conditions, which will lead to a major step forward in bioconjugation and chemical biology.

**Fig. 1:**
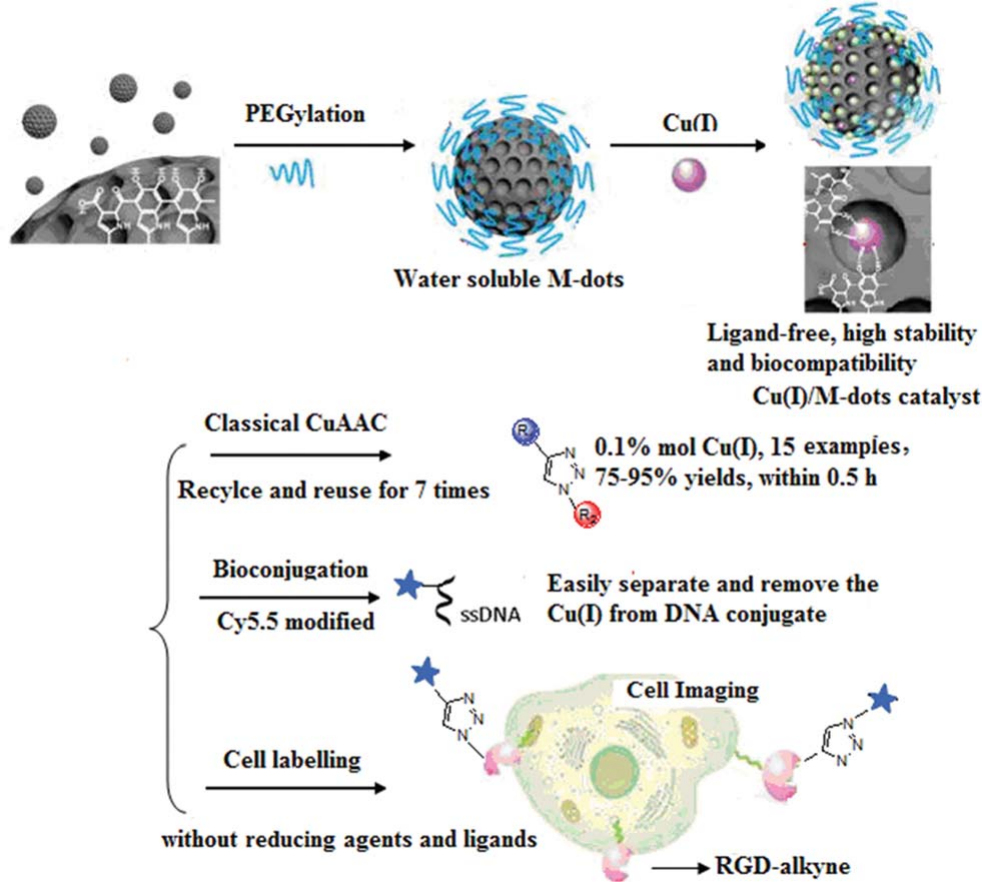
The preparation of Cu(i)/M-dots heterogeneous catalyst and its applications in classic CuAAC reactions, bioconjugation and cell labeling.

Considering the merits of M-dots, we envision that loading Cu(i) into M-dots can obtain a ligand-free, water-soluble, biocompatible and recyclable Cu(i) catalyst. Moreover, intrinsic nitrogen groups presented in M-dots can coordinate the metal ions and the resulting Cu(i)/M-dots can greatly accelerate the kinetics of Cu(i)-catalyzed reactions and stabilize the Cu(i) oxidation state. As a proof of concept, we prepared a novel Cu(i)/M-dot catalyst and demonstrated its excellent properties when applied in the CuAAC reaction, DNA bioconjugation and cell labeling (Fig. 1). To the best of our knowledge, reports of metal-based heterogeneous catalysis in biological applications are still scarce compared to their homogeneous counterparts.^24^,^25^

## Results and discussion

Fig. 1 schematically illustrates the procedure for preparing the Cu(i)/M-dots heterogeneous catalyst. The M-dots were synthesized from commercial melanin granules according to a previous report.^12^ The average sizes of M-dots were ∼7 nm measured by transmission electron microscopy (TEM) (ESI Fig. S1A†). The hydrodynamic diameters of M-dots were ∼7.45 nm by dynamic light scattering (DLS), and the zeta potential of M-dots was –2.2 mV (ESI Table S1†). The ^1^H-NMR of M-dots in D_2_O display a characteristic signal at 3.58 ppm due to the hydrogen atoms of the PEG chains (ESI Fig. S1B†). The molecular weight of the M-dots was about 86 kDa determined by MALDI-TOF MS (ESI Fig. S1C†). All these data confirm the successful preparation of M-dots. In our previous work, PEG encapsulation was demonstrated to enhance biocompatibility, metal loading and water-solubility.^12^

**Fig. 2:**
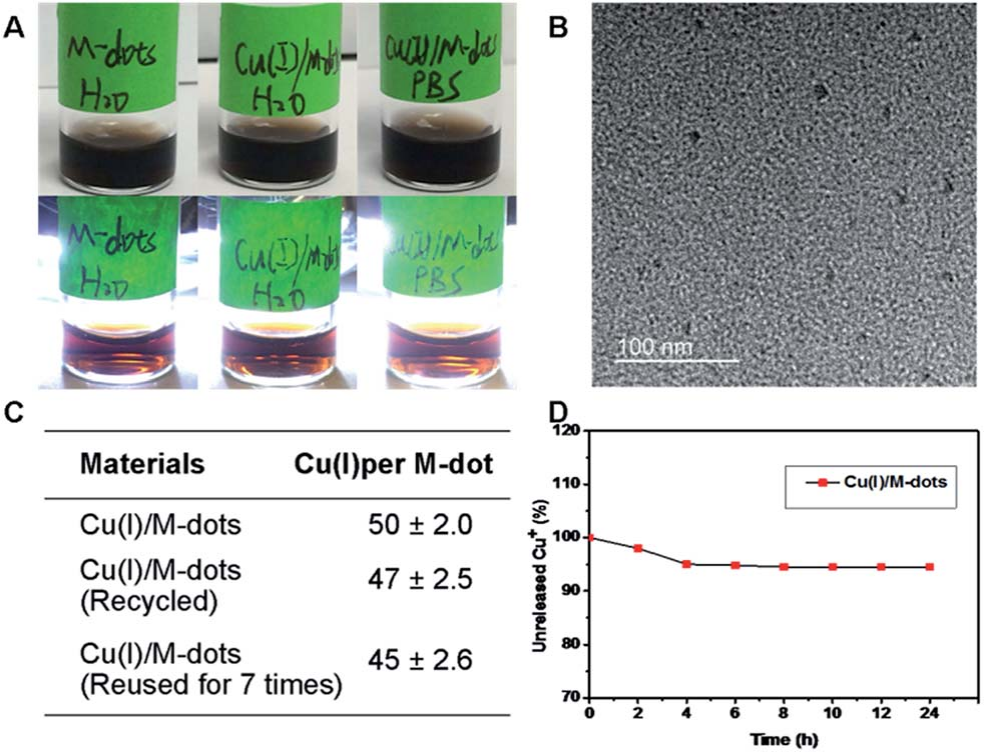
Characterization of the physical properties of Cu(i)/M-dots. (A) From left to right: photo of (1) M-dots stored in water, (2) Cu(i)/M-dots stored in water, and (3) Cu(i)/M-dots stored in PBS without (top) or with (bottom) flash light illumination. (B) TEM of Cu(i)/M-dots. (C) Inductively coupled plasma mass spectrometry (ICP-MS) results of the number of Cu(i) attached to one M-dot. (D) Stability study of Cu(i)/M-dots in PBS (pH = 7.4).

Encouraged by the vast applications of CuAAC chemistry, the preparation of Cu(i)/M-dots was undertaken (see ESI†). The Cu(i)/M-dots were purified using a centrifugal filter (MWCO = 30 kDa). After purification, the fresh Cu(i)/M-dots catalyst maintained good water and phosphate buffer (PBS)-solubility without any observed precipitation (Fig. 2A). A TEM image indicates that Cu(i)/M-dots were monodispersed and homogeneous in water (Fig. 2B). The Cu(i)/M-dots exhibited good Cu(i) loading capacities. The number of freshly prepared Cu(i) per single M-dot was determined to be 50 ± 2.0 (mean ± SD, Fig. 2C) according to the inductively coupled plasma mass spectrometry (ICP-MS) results. After Cu(i)-chelation, the size of the Cu(i)/M-dots increased to ∼9 nm and its zeta-potential went from –2.2 mV to +5.1 mV due to the introduction of positive Cu(i) on the M-dots surface (ESI Table S1†). The presence of Cu(i) on the M-dots platform was confirmed by X-ray photoelectron spectroscopy (XPS) (ESI Fig. S2†). The binding energy peaks at 931.6 and 952.8 eV can be attributed to those characteristic of Cu(i).^26^ The possible chelating mechanisms of Cu(i) with M-dots is shown in Fig. S3.† A stability assay of Cu(i)/M-dots in PBS indicates the high stability of the chelating platform (Fig. 2D). Moreover, we investigated the biocompatibility of the Cu(i)/M-dots compared to the classic Cu(i)/THPTA complex (abbreviation THPTA: [tris(3-hydroxypropyltriazolylmethyl)amine], a widely used and water-soluble ligand for CuAAC) on two cell lines: mouse embryonic fibroblast cell line (NIH3T3) and human glioblastoma cell line (U87MG) with different Cu(i) concentrations (50, 100, 200 µM) and different incubation times (1, 12, 24, 48 h). For a short incubation time (1 h), both Cu(i)/M-dots and Cu(i)/THPTA systems were non-toxic to two cell lines. However, at longer incubation times (12, 24, and 48 h) an increased cytotoxicity was observed at all copper concentrations (50 µM to 200 µM) for Cu(i)/THPTA. In contrast, no toxicity of Cu(i)/M-dots was observed according to dimethylthiazolyl-diphenyltetrazolium (MTT) assays for all time points (Fig. S4 and S5†). These results clearly demonstrate that Cu(i)/M-dots show high biocompatibility and are promising for further biological applications.

Ascorbate-driven, Cu(i)-induced hydroxyl radical production is a major issue in CuAAC bioconjugate chemistry.^13^ Therefore, we investigated the free radical quenching ability of M-dots in a model reaction with 3-coumarin carboxylic acid (3-CCA, hydroxyl radical detector) in the presence of Cu(ii)/ascorbate.^27^ 3-CCA was stable in the presence of CuSO_4_ or ascorbate alone in PBS, but the combination of the two induced the oxidation of approximately 50% of 3-CCA to 7-OH CCA after 20 h (ESI Fig. S6†). Therefore, M-dots not only functioned as a traditional support for a heterogeneous catalyst, but also scavenged radical molecules to reduce the toxicity induced by Cu(i), which can greatly expand its biological application.

The relative reactivity of M-dots-based heterogeneous catalysts, Cu(i)/M-dots and Cu(ii)/M-dots with or without ascorbate was evaluated using a coumarin-based fluorogenic probe which enabled us to monitor the reaction by simple fluorescence measurements of the corresponding triazole product (ESI Fig. S7,† ex = 320 nm; em = 400 nm).^28^ As expected, Cu(i)/M-dots afforded high yields, and the kinetics was improved by increasing the amount of Cu(i)/M-dots. A significantly lower yield (<5%) was observed under Cu(ii)/M-dots treatment. However, a high yield was obtained by the addition of ascorbate to the Cu(ii)/M-dots system. Thus, the function of M-dots appears to be crucial not only for maintaining the stability of the Cu(i) catalyst, but also for greatly accelerating the reaction.

**Fig. 3:**
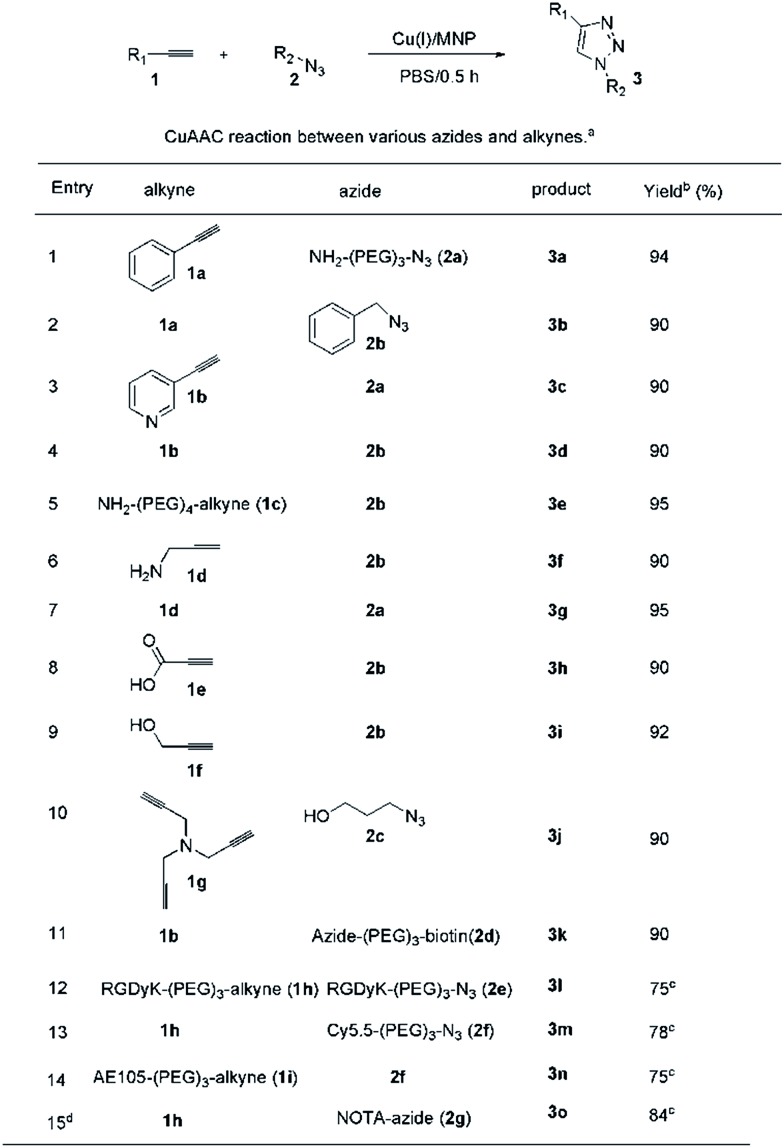
CuAAC reactions between various azides and alkynes using 0.1 mol% Cu(i)/M-dots in PBS for 0.5 h. (a) Conditions: 0.1 mmol alkyne, 0.12 mmol azide and 2 nmol Cu(i)/M-dots (containing 0.1% mol Cu^+^). (b) Isolated yields calculated on the starting alkyne. (c) The products were purified byHPLC. 1,4,7-Triazacyclononane-triacetic acid is abbreviated as NOTA.

Based on these promising results, the scope of the CuAAC was examined using various substrates (Fig. 3). To our delight, the Cu(i)/M-dots system was efficient for each substrate (only 0.1% mol Cu^+^), leading to yields of about 90% in the absence of ascorbate and ligands (entries 1–10). These result confirm that Cu(i) was stabilized within the M-dots thus avoiding the use of a reducing agent. Moreover, the construction of biomolecules of medical interest was also explored (entries 11–15). Derivatives of biotin **3k** and dimeric RGD **3l** were obtained in good yields (entry 11–12). The imaging precursors/probes (Cy5.5-RGD (abbreviation Cy5.5: Cyanine5.5, **3m**), Cy5.5-AE105 (**3n**) and NOTA-RGD (abbreviation NOTA: 1,4,7-triazacyclononane-triacetic acid, **3o**)) were also prepared in 78%, 75% and 84% yields, respectively (entries 13–15). **3o** was further efficiently labelled with a PET radionuclide, ^64^Cu, in 100% radiolabelling yield (ESI Fig. S8†). This indicates that no free Cu(i) escaped from the M-dots, which further confirms the stability of the Cu(i)/M-dots catalyst under CuAAC conditions.

Further experiments showed that the Cu(i)/M-dots was a robust and recyclable catalyst. It was recycled by centrifugation and used for seven cycles without loss of catalytic activity (ESI Table S2,† entries 1–7). Quantitative analysis of the product from each cycle by ICP-MS revealed no detectable leaching of Cu(i). TEM and photo images of reused Cu(i)/M-dots reveal that the structure of the catalyst remained intact and stable (ESI Fig. S9†). Moreover, the Cu(i)/M-dots system also efficiently drives the three-component click reaction of alkyl halides, sodium azide and alkynes with good recyclability (ESI Table S3 and Fig. S10†).

**Fig. 4:**
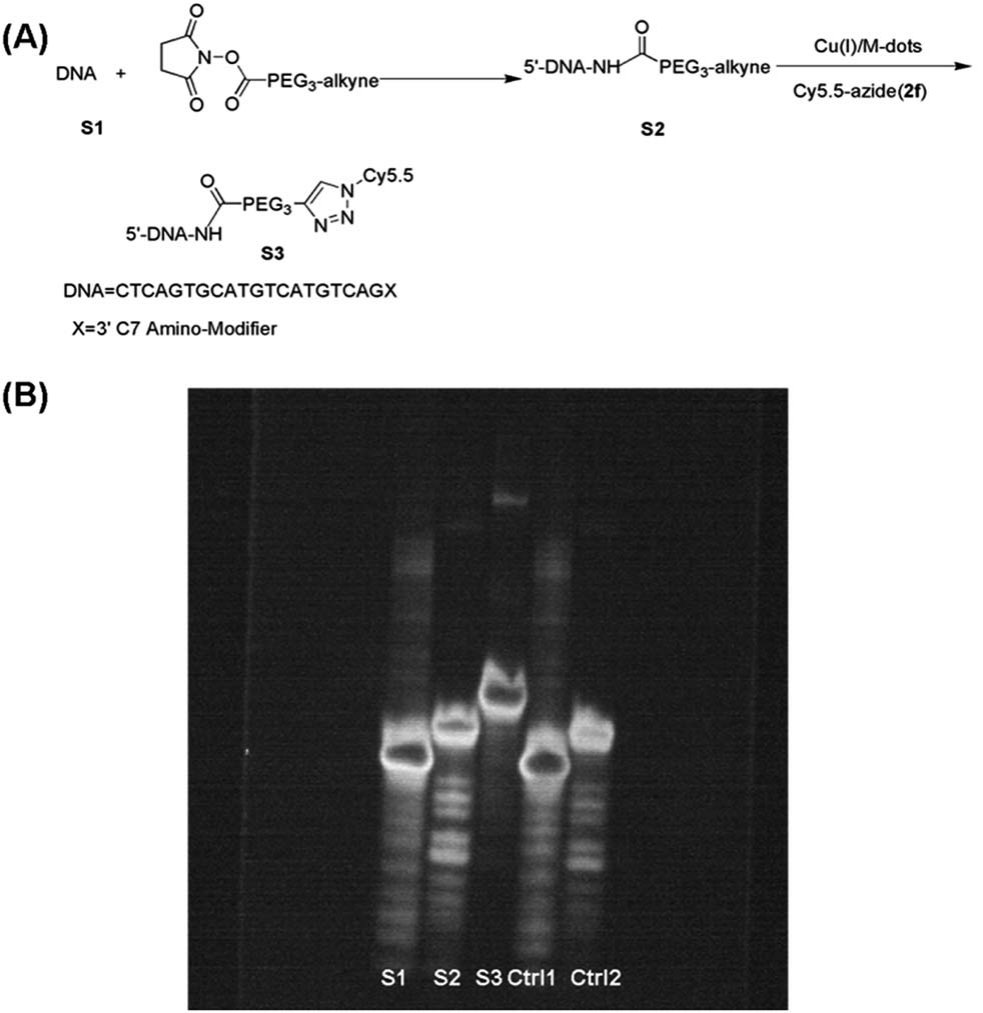
Demonstration of DNA bioconjugation by the CuAAC reaction: (A) reaction scheme. (B) 16% denaturing polyacrylamide (PAGE) gel electrophoresis of **S1** (lane 1), **S2** (lane 2), **S3** (lane 3), Control 1 (lane 4, **S1** incubated with **2f** and Cu(i)/M-dots for DNA conjugation and then separated by centrifugal filtration (MWCO = 10 kDa) for analysis) and Control 2 (lane 5, **S2** incubated with a **2f** and M-dots without copper loading for DNA conjugation and then separated by centrifugal filtration (MWCO = 10 kDa) for analysis) at room temperature and imaged under UV.

The utility of the Cu(i)/M-dots for bioconjugation was tested on an alkyne derivatised DNA strand with an azide fluorophore in low concentrations. The 3′-position modified oligonucleotide (**S1**, 5 nmol) in 20 µL of water was reacted with an excess of NHS ester **V1** (50 equivalents) in 30 µL of water at room temperature for 12 h. Unreacted **V1** was removed by a centrifugal filter (3 kDa), and then lyophilized to obtain alkyne-labelled DNA **S2**. A click reaction of **S2** (20 µM) was then performed with Cy5.5-azide (**2f**, 80 µM) mediated by 2 µM Cu(i)/M-dots (containing about 100 µM Cu^+^) in 0.1 M PBS at pH 7 for 2 h (Fig. 4A). Unreacted Cy5.5 dye was removed by centrifugal filter (MWCO = 3 kDa). Then, the resulting fluorescent DNA **S3** was separated from the catalyst by centrifugal filter (MWCO = 10 kDa). **S1**, **S2**, **S3** and two control groups were loaded on a 16% denaturing PAGE gel for electrophoresis (15 mA, 3 h and 1× TBE buffer). Then, the DNA PAGE gel was imaged under 365 nm (UVP, UV imager) revealing that DNA bioconjugation was efficiently carried out only in the presence of Cu(i)/M-dots (Fig. 4B). In addition, MALDI-TOF MS showed an expected molecular weight for the corresponding cycloadduct (ESI Fig. S11†). Unlike traditional homogeneous Cu(i)/ligand/NaAsc systems, the Cu(i)/M-dots did not require a reducing agent, which is known to cause undesirable side effects such as covalent modification and potential aggregation of proteins.^29^,^30^ Moreover, the Cu(i)/M-dots system can easily be separated from biomolecules and can thus solve the problem of contamination from the residual Cu(i) catalyst.

**Fig. 5:**
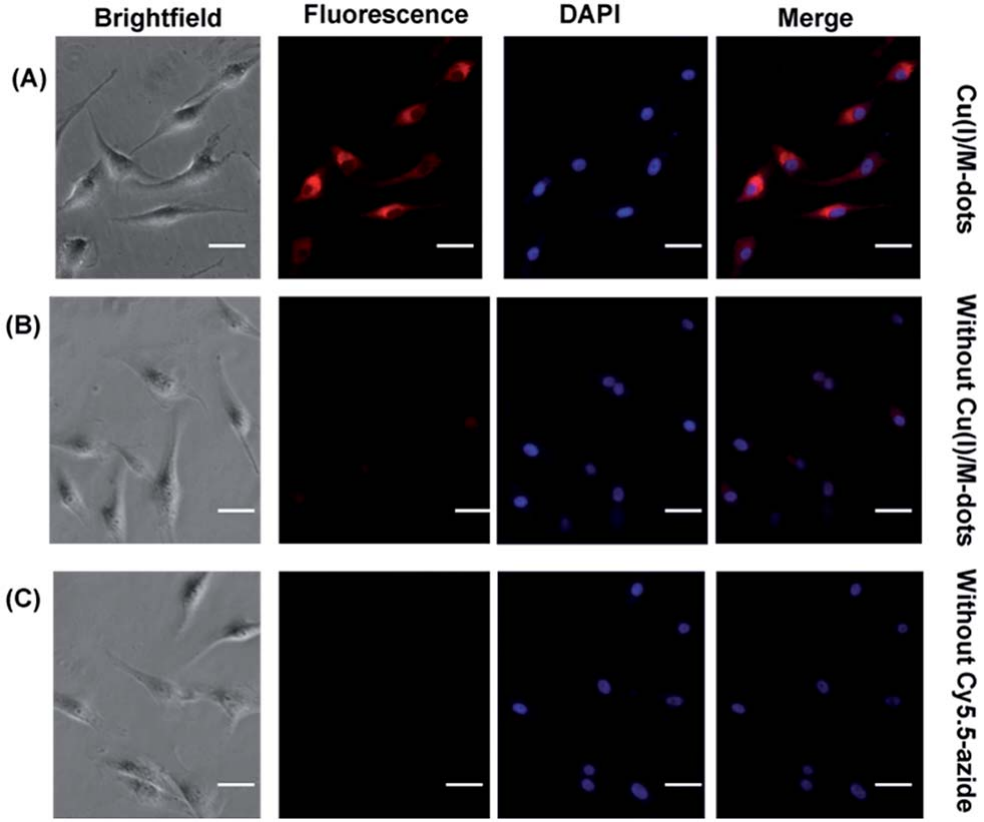
Cell labelling with Cu(i)/M-dots for fluorescence microscopy imaging. The scale bar represents 40 µm. (A) U87MG cells were first treated with RGD-alkyne (10 µM) for 30 min at 37 °C, followed by incubation with Cy5.5 azide (30 µM) and 4 µM Cu(i)/M-dots (containing 200 µM Cu^+^) for 60 min at 37 °C. After cells were fixed with 4% PFA, DAPI staining was performed. (B) Control experiment without adding Cu(i)/M-dots for incubation. (C) Control experiment without adding Cy5.5 azide for incubation.

To assess the capability of the Cu(i)/M-dots for cell labelling, we used Cy5.5 azide and RGD-alkyne in combination with Cu(i)/M-dots for imaging of the integrin α_V_β_3_ receptor. Human glioblastoma U87MG cells were selected because of their high level of integrin α_V_β_3_ expression.^31^ The U87MG cells were first labelled with RGD-alkyne (**1h**, 10 µM) to cap the alkyne functional group on the integrin protein due to the strong binding ability of the RGD peptide with the α_V_β_3_ integrin receptor.^32^ After several washing steps to remove any unbound alkyne, Cy5.5 azide (**2f**, 30 µM) and 4 µM Cu(i)/M-dots (containing about 200 µM Cu^+^) were added to the cell culture medium for 60 min at 37 °C. After the click reaction for labelling live cells and several PBS washing steps to remove Cy5.5 azide and Cu(i)/M-dots, the U87MG cells were fixed with 4% paraformaldehyde (PFA) and further stained with 4′,6-diamidino-2-phenylindole (DAPI) to reveal the location of the nuclei. The cells were then imaged using fluorescence microscopy (Axiovert 200M fluorescence microscope). Control studies were performed in the absence of Cy5.5 azide or Cu(i)/M-dots. The recorded microscopy images reveal a strong fluorescent signal of the U87MG cells incubated with RGD-alkyne, Cy5.5 azide and Cu(i)/M-dots (Fig. 5A), whereas much weaker signals are observed for the control groups (Fig. 5B and C). The results clearly indicate that the Cu(i)/M-dots enable efficient performing of the CuAAC on the cell membrane.

## Conclusions

In conclusion, we reported M-dots as an effective nanoplatform for preparing a ligand-free and water-soluble heterogeneous catalyst, Cu(i)/M-dots. Cu(i)/M-dots is proven to be a high performance, biocompatible and recyclable catalytic system for CuAAC and bioconjugation without any Cu(i) toxicity. M-dots can serve as an novel metal-loading recyclable catalyst for various applications in bioconjugation and chemical biology.

## Supplementary Material

Supplementary information
