# Supplementary material for: Recyclable Cu(i)/melanin dots for cycloaddition, bioconjugation and cell labelling
Source: Chem Sci. 2016 May 20;7(9):5888–92. doi: 10.1039/c6sc01536k (PMC6024301; doi:10.1039/c6sc01536k)

Electronic Supplementary Material (ESI) for Chemical Science.
This journal is © The Royal Society of Chemistry 2016

Supporting Information

Recyclable Cu(I)/Melanin Dots for Cycloadditions, Bioconjugation and Cell Labeling

Yao Sun,[†,‡] Su Hyun Hong,[†] Xiaowei Ma,[†] Kai Cheng,[†] Jing Wang,[†] Zhe Zhang,[†] Meng Yang,[#] Yuxin Jiang,[#] Xuechuan Hong,*[,‡] and Zhen Cheng*[,†]

[†] *Molecular Imaging Program at Stanford (MIPS), Bio-X Program, and Department of Radiology, Canary Center at Stanford for Cancer Early Detection, Stanford University, California, 94305-5344*

[‡] *State Key Laboratory of Virology, Key Laboratory of Combinatorial Biosynthesis and Drug Discovery (Wuhan University), Ministry of Education, Wuhan University School of Pharmaceutical Sciences, Wuhan 430071, People's Republic of China*

#Chinese Academy of Medical Science, Peking Union Medical College Hospital, Department of Ultrasound, Beijing, 100730, People's Republic of China

*Correspondence should be sent to: zcheng@stanford.edu or xhy78@whu.edu.cn

Table of Contents

		Page
1.	Supplementary Figures S1-S11	S3-13
2	Table S1-S3	S14-16
3	General method	S17-22
4	Chemical synthesis and characterization	S22-35
5	^{1}H and ^{13}C NMR	S36-48

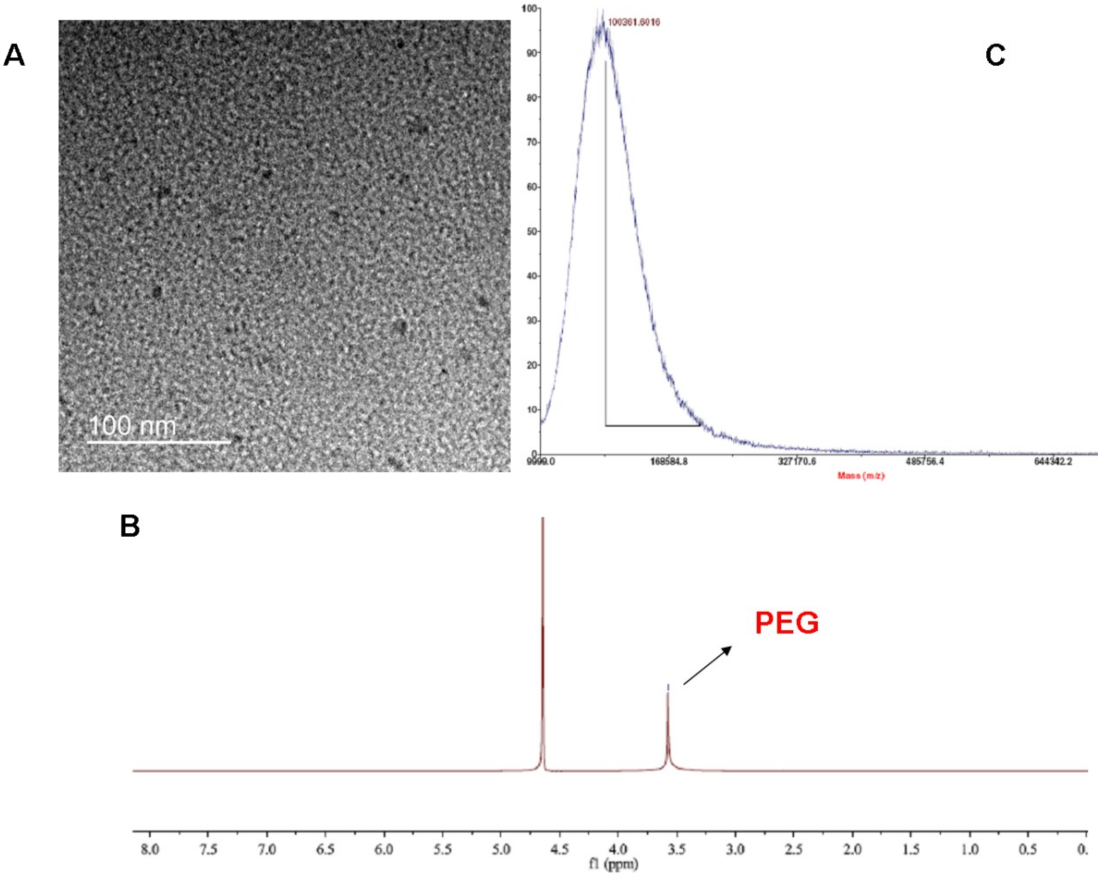

Figure S1. (**A**) TEM image of M-dots; (**B**) ^{1}H-NMR spectra of M-dots; (**C**) MALDI-TOF-MS of M-dots.

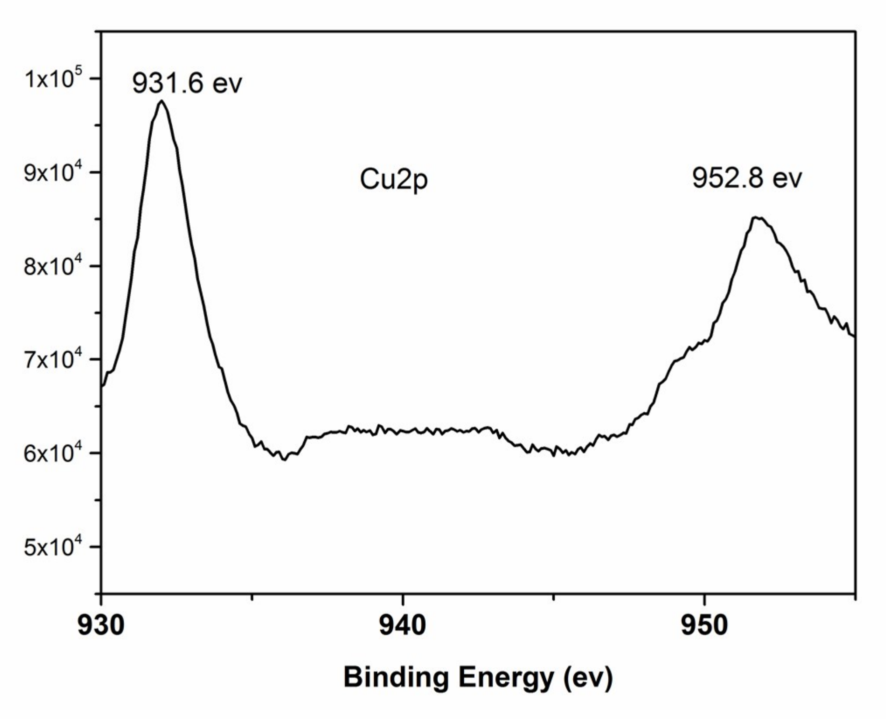

Figure S2. XPS of Cu(I)/M-dots.

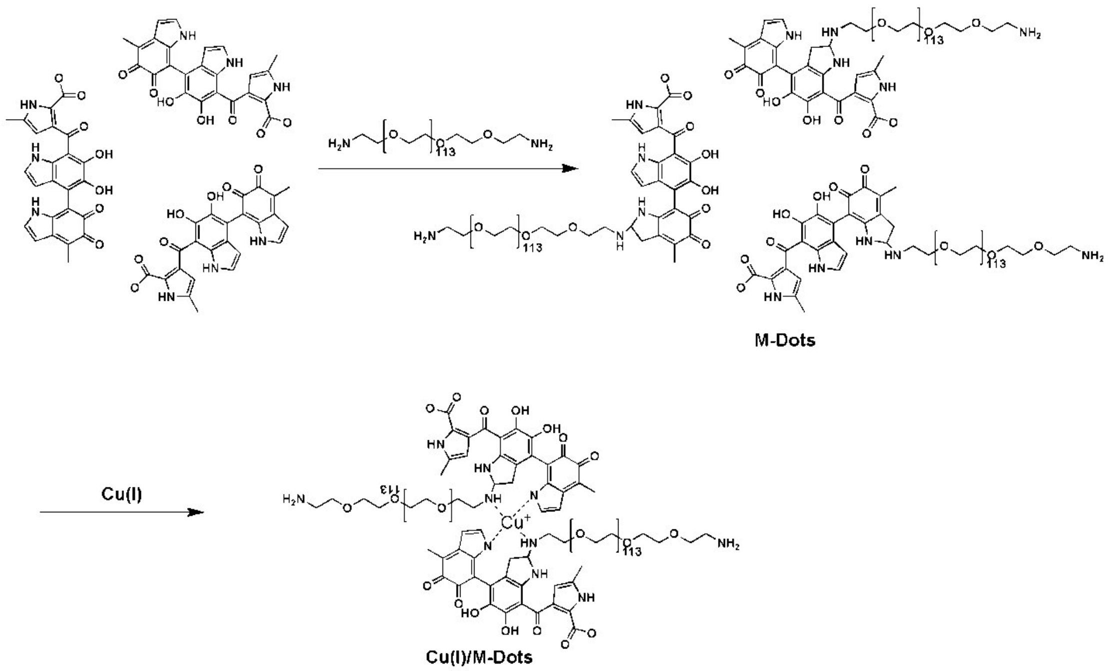

Figure S3. Synthetic route for Cu(I)/M-dots and the possible chelating mechanism of Cu(I) and M-dots

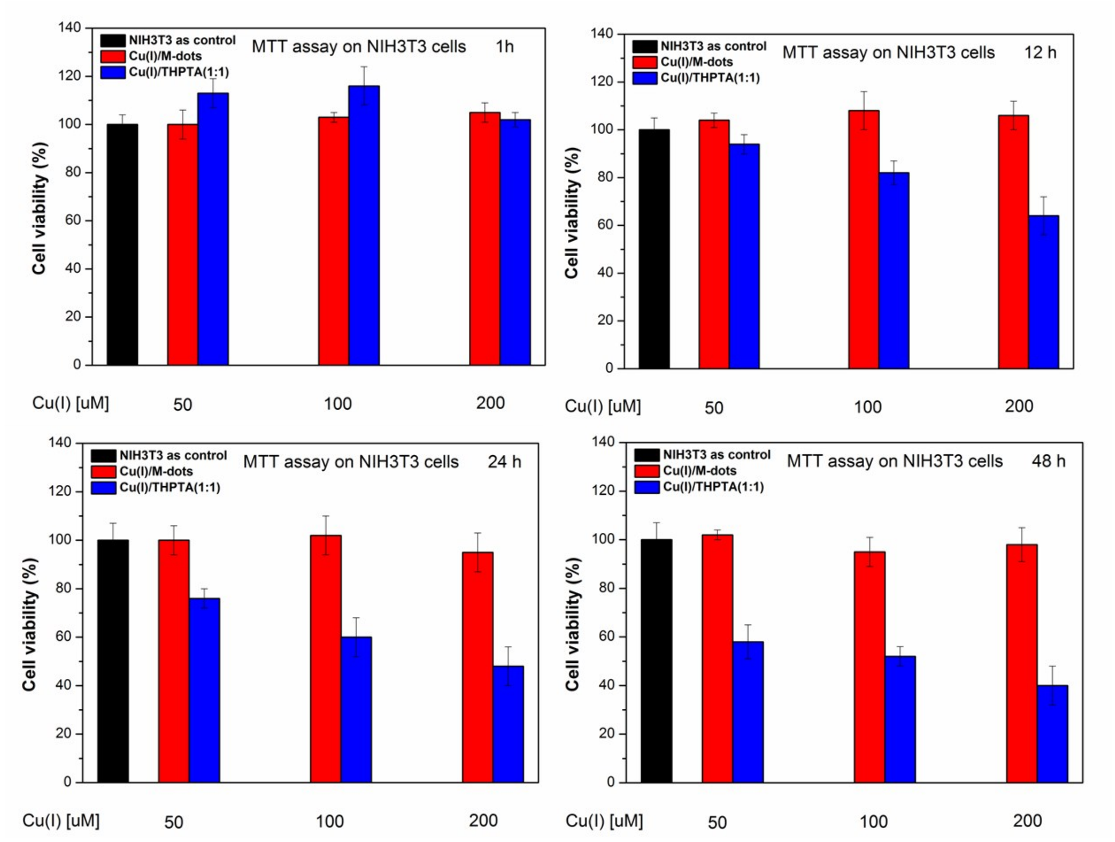

Figure S4. Viability of NIH3T3 cells in the presence of Cu(I)/M-dots and Cu(I)/THPTA with different Cu(I) concentrations 50, 100, 200 μM after 1h, 12h, 24 h and 48 h incubation at 37°C. Abbreviation THPTA: [tris(3-hydroxypropyltriazolylmethyl)amine].

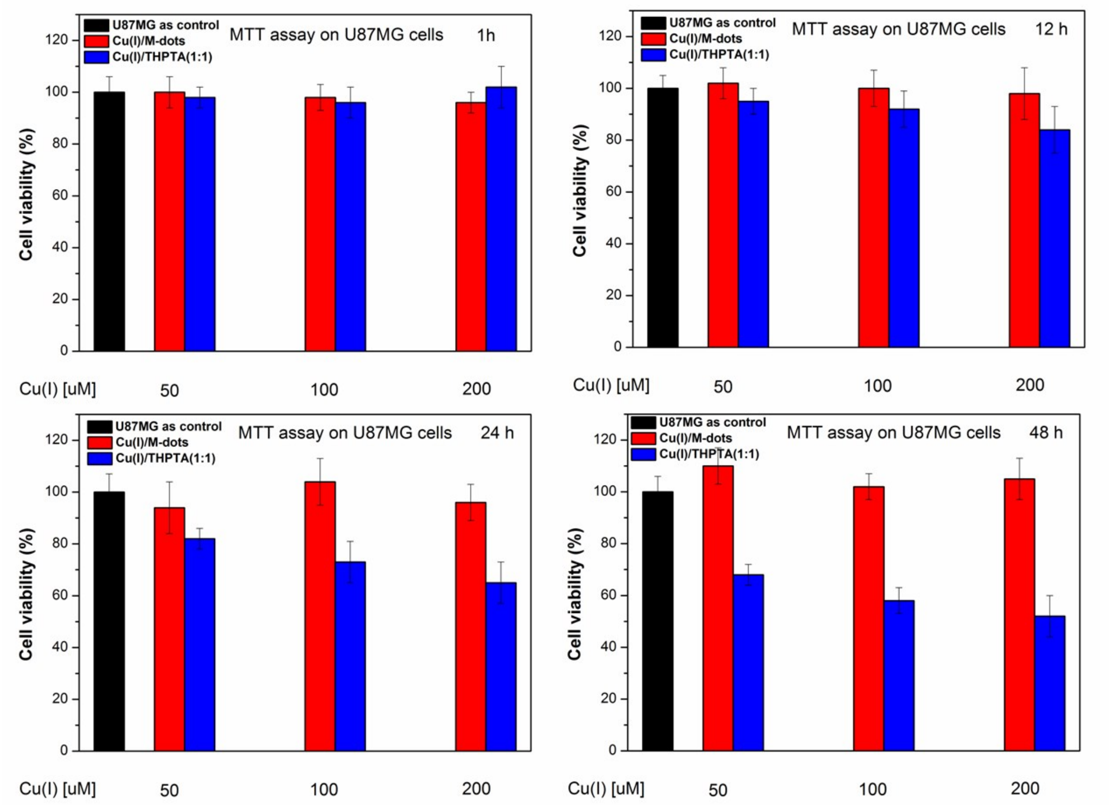

Figure S5. Viability of U87MG cells in the presence of Cu(I)/M-dots and Cu(I)/THPTA with different Cu(I) concentrations 50, 100, 200 µM after 1h, 12h, 24 h and 48 h incubation at 37°C.

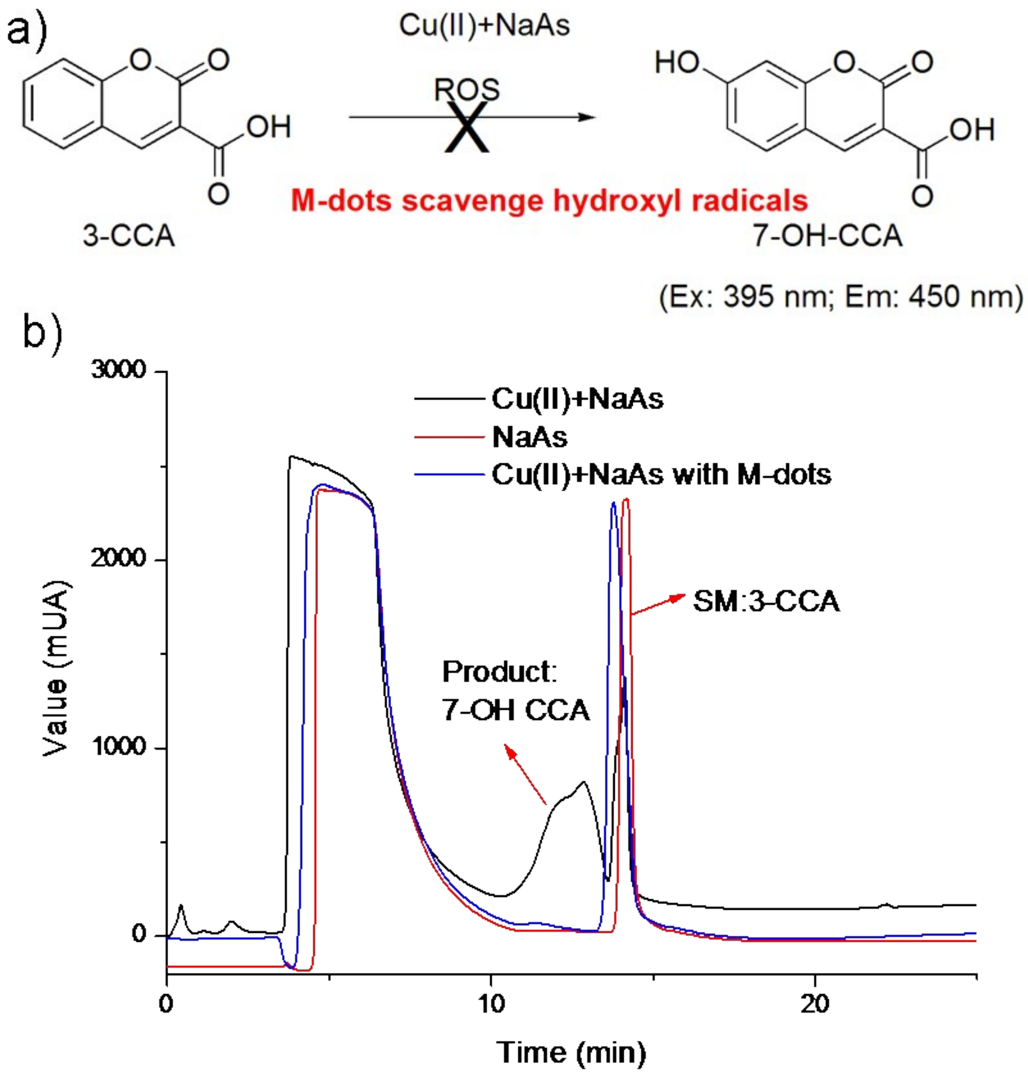

Figure S6. **(a)** The pathway of ascorbate-mediated, Cu(I)-induced hydroxyl radical production was scavenged by M-dots; (b**)** A fluorogenic ROS reporter 3-CCA (3-coumarin carboxylic acid, 100 μM) was used for the ROS detection in vitro. The HPLC spectra monitored this radical reaction after 12 h. The condition of $CuSO_4$ (100 μM) and ascorbate (5 mM) could produce ROS product 7-OH CCA (black line), while with M-dots (100μM), no ROS product generates in the presence of $CuSO_4$/ascorbate (blue line). As a control, no ROS signal generate in the presence of ascorbate (red line).

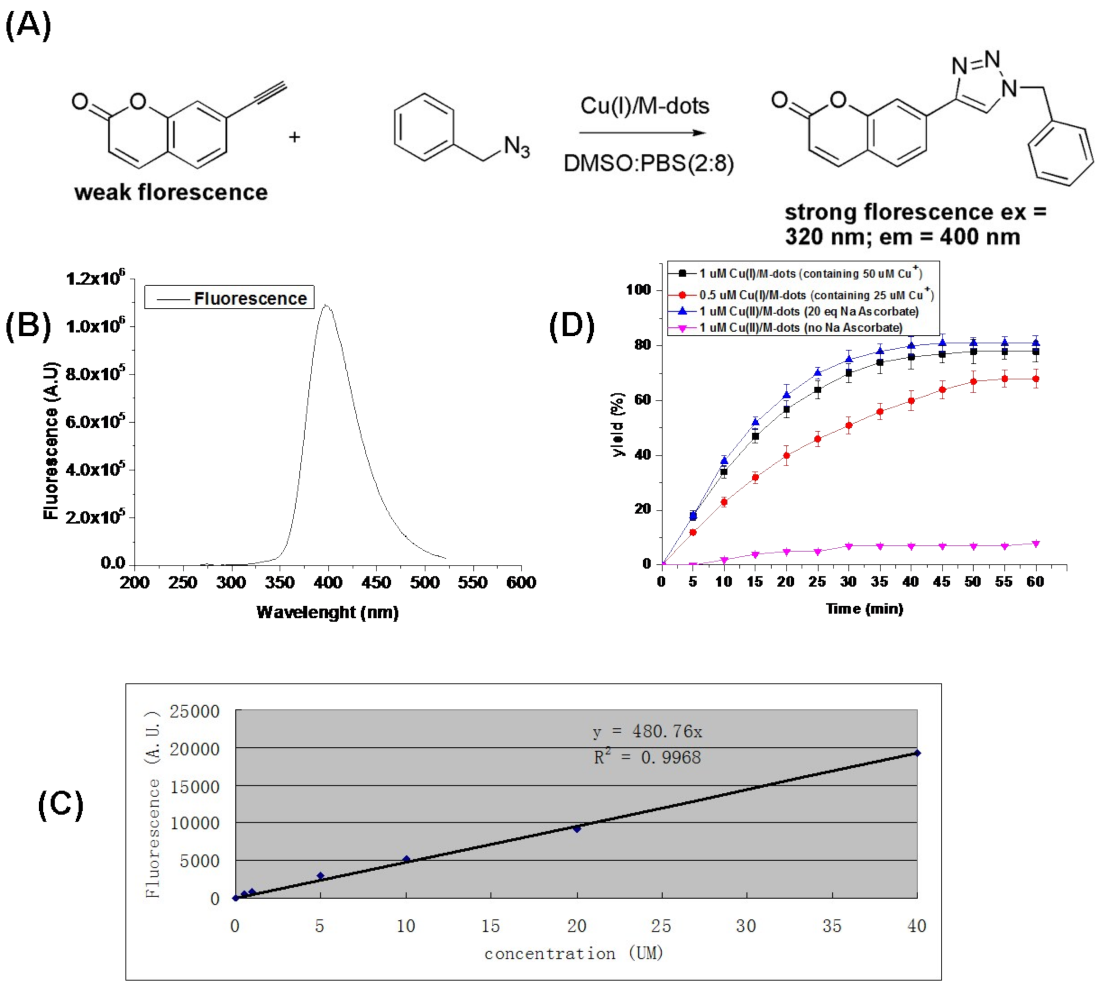

Figure S7. (A) The fluorogenic CuAAC reaction of coumarin-based fluorogenic probe and benzyl azide results in a highly fluorescent triazole product. (B) The fluorescence emission spectrum; (C) The standard curve that correlates fluorescence signal to triazole concentrations; (D) Comparison of CuAAC kinetics: Cu(I)/M-dots, Cu(II)/M-dots and Cu(II)/M-dots with sodium ascorbate (20 eq).

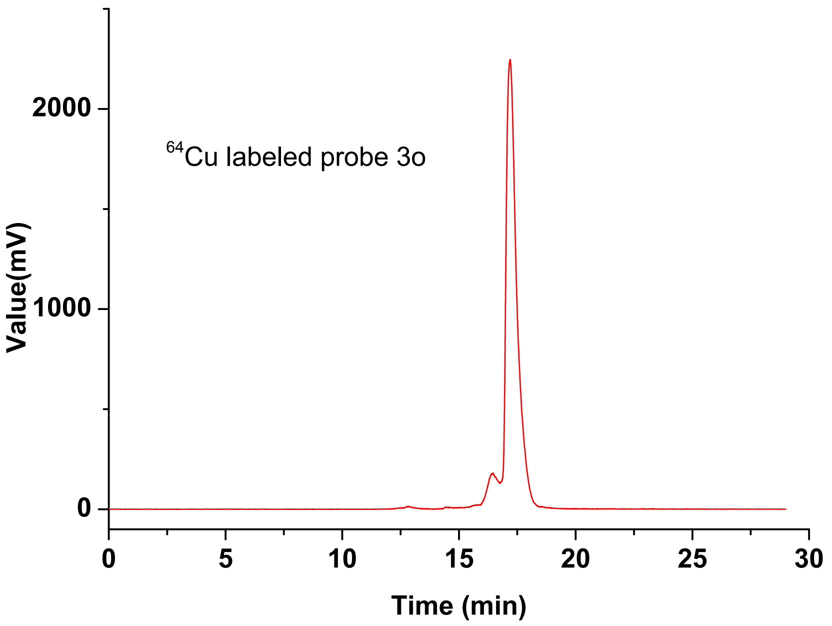

Figure S8. Radio-HPLC chromatogram of ^{64}Cu radiolabeled 3o.

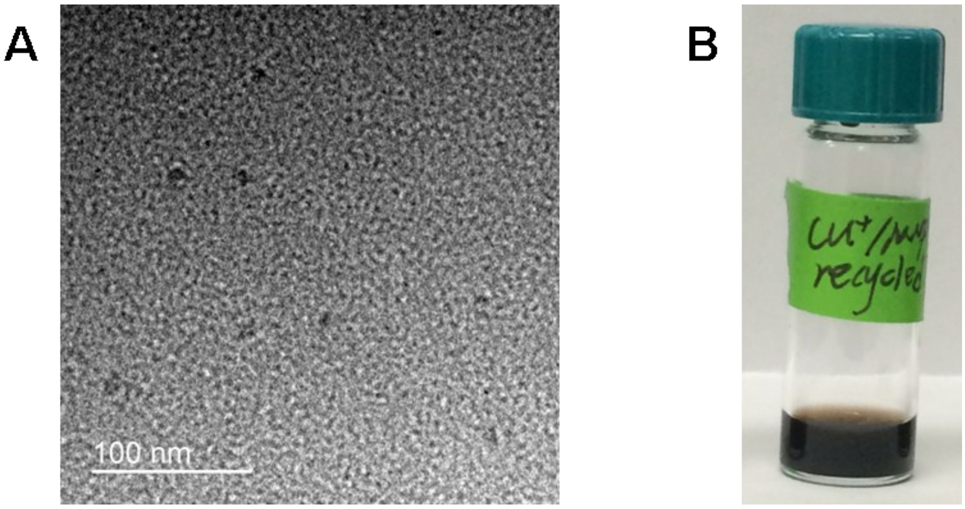

Figure S9. (A) TEM image of recycle Cu(I)/M-dots catalyst; (B) photo image of recycle Cu(I)/M-dots catalyst.

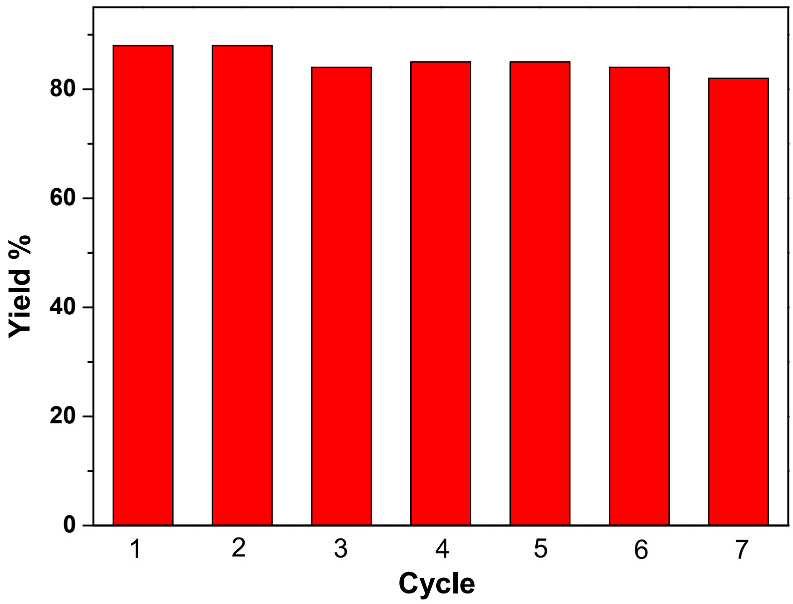

Figure S10. Recycling study of Cu(I)/M-dots catalytic system for Three-Component Cyclization.

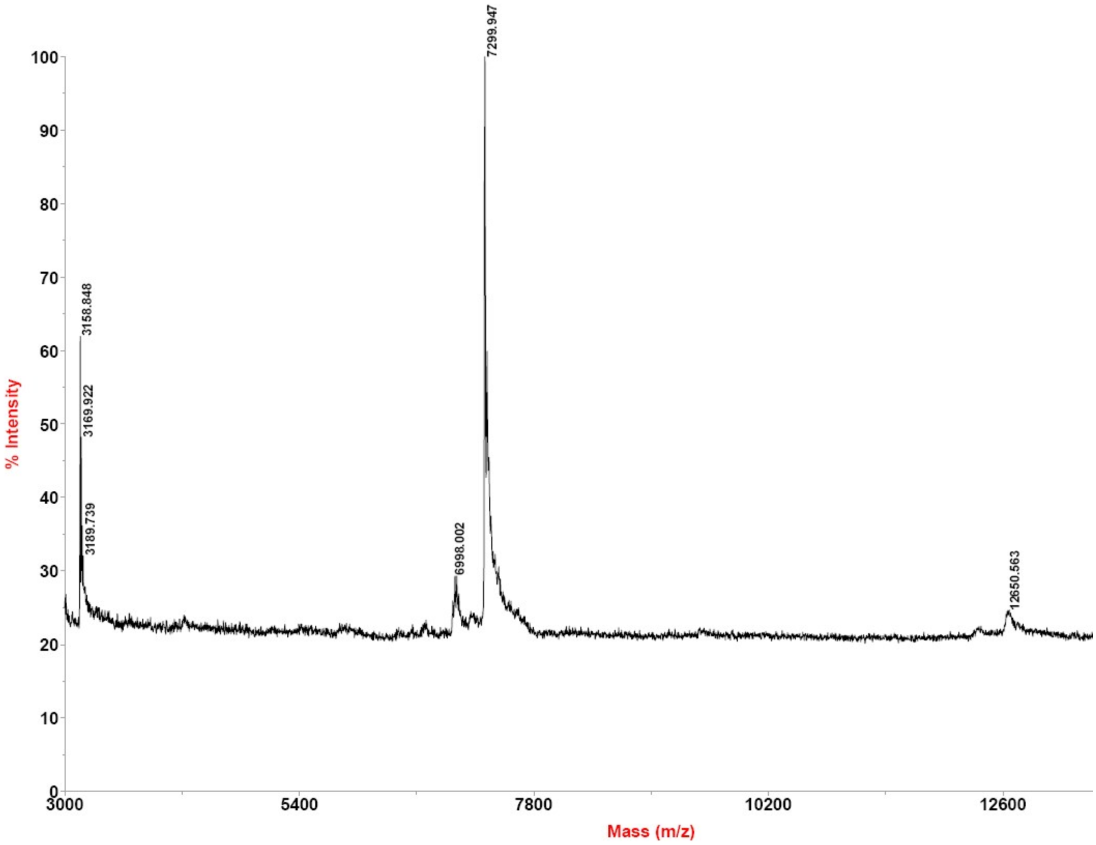

Figure S11. MALDI-TOF-MS of DNA conjugate.

M-dots	Diameter (nm)	Zeta potential (mV)
M-dots	7.45 ± 1.22	-2.2 ± 2.43
Cu(I)/M-dots	8.55 ± 1.87	+5.1 ± 3.26

Table S1. The data of hydrodynamic sizes and zeta potentials of M-dots and Cu(I)/M-dots in aqueous solution. The hydrodynamic sizes of M-dots and Cu(I)/M-dots were measured by dynamic light scattering (DLS) instrument using a 90 Plus particle size analyzer (Malvern, Zetasizer Nano ZS90). Zeta potential was measured using a zeta potential analyzer (Malvern, Zetasizer Nano ZS90).

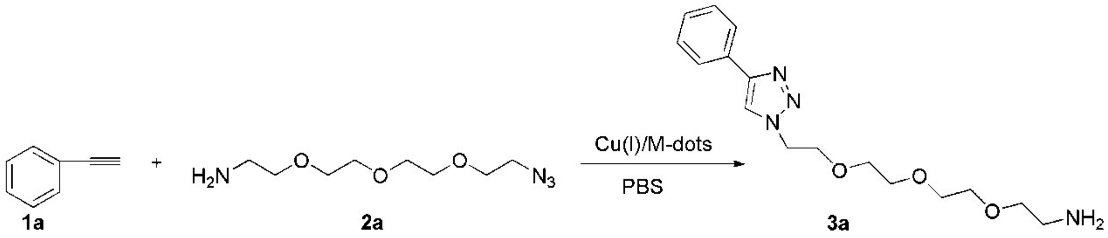

Entry	alkyne	Cycle[a]	Yield (%)
1	10.2 mg (100 μmmol)	1st	88
2	10.2 mg (100 μmmol)	2nd	85
3	10.2 mg (100 μmmol)	3rd	85
4	10.2 mg (100 μmmol)	4	84
5	10.2 mg (100 μmmol)	5	85
6	10.2 mg (100 μmmol)	6	80
7	10.2 mg (100 μmmol)	7	82

a:Cu(I)/M-dots was removed by centrifugal-filtare (MWCO=30K), and washed by water (3 times) for directly using in the next reaction

Table S2. The recycle study of Cu(I)/M-dots catalytic system for two component [3+2] cycloaddition reaction.

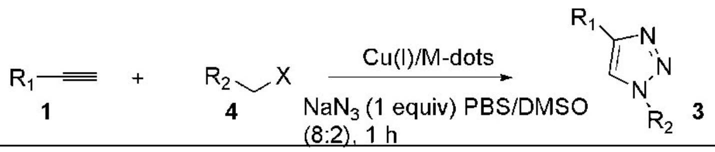

Entry[a]	alkyne	alkyl halides	product	Yield[b] (%)
1	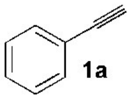 1a	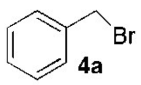 4a	3b	88
2	1a	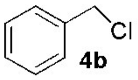 4b	3b	85
3	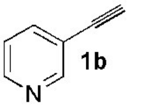 1b	4a	3c	78
4	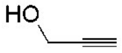 1f	4a	3i	90
5	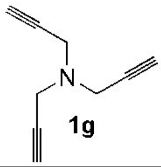 1g	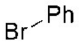 4c	3p	67

Conditions: 0.1 mmol alkyne, 0.12 mmol Halide and 0.12 mmol sodium azide, 2 nmmol Cu(I)/M-dots in 1mL (containing 0.1% mol Cu^+)

Table S3. Three-Component cyclization of alkyl halides, sodium azide, and alkynes.

General methods

All chemicals were purchased from commercial sources (such as Aldrich). NOTA-Azide was purchased from AREVA Med. Azido-PEG3-NHS and Alkyne-PEG3-NHS were purchased from Conju-Probe. Cy5.5 dye was purchased from Lumiprobe. RGDyK was purchased from peptide international. Dimethylthiazolyl-diphenyltetrazolium (MTT) was purchased from Biotium. Phosphate buffered saline (PBS) was purchased from Gibco. Amine-PEG5000-Amine (NH_2-PEG_{5000}-NH_2, 5kDa) was purchased from Laysan Bio. GelRed Nucleic Acid Gel Stain was purchased from Biotium. Tris base, Boric acid, Na_2EDTA, Acrylamide, bisacrylamide, Urea, ammonium persulfate (APS) and tetramethylethylenediamine (TEMED) purchased from Thermo Fisher. The ^1H and ^{13}C NMR spectra were acquired on a Bruker 400 MHz magnetic resonance spectrometer. Data for ^1H NMR spectra are reported as follows: chemical shifts are reported as *δ* in units of parts per million (ppm) relative to chloroform-d (δ 7.26, s); multiplicities are reported as follows: s (singlet), d (doublet), t (triplet), q (quartet), dd (doublet of doublets), m (multiplet), or br (broadened); coupling constants are reported as a *J* value in Hertz (Hz); the number of protons (n) for a given resonance is indicated nH, and based on the spectral integration values. MALDI-TOF-MS spectrometric analyses were performed at the Mass Spectrometry Facility of Stanford University. HPLC was performed on a Dionex HPLC System (Dionex Corporation) equipped with a GP50 gradient pump and an in-line diode array UV-Vis detector. A reversed-phase C18 (Phenomenax, 5 µm, 4.6 × 250 mm, 5 µm, 10 × 250 mm or 21.2 × 250 mm) column was used for analysis and semi-preparation. UV absorbance of the probe was recorded on an Agilent 8453 UV spectrophotometer. Fluorescence was recorded on a Fluoromax-3 spectrafluorometer (Jobin Yvon). The hydrodynamic sizes of M-dots and Cu(I)/M-dots were measured by dynamic light scattering (DLS) instrument using a 90 Plus particle size analyzer (Malvern, Zetasizer Nano ZS90). Zeta potentials were measured using a zeta potential analyzer (Malvern, Zetasizer Nano ZS90). Transmission electron microscopy (TEM) images were recorded on a JEOL 2010 transmission electron microscope. X-ray photoelectron spectra was recorded by using Thermo ESCALAB 250Xi X-ray photoelectron spectrometer. The Cu ions analyses were

performed using inductively coupled plasma mass spectrometer (ICP-MS, Thermo Scientific Xseries 2 Quadrupole). PAGE gel was visualized by a UV transilluminator (UVP). The cells were imaged using fluorescence microscopy (Axiovert 200M fluorescence microscope).

Preparation of M-dots

Tyrosine-derived synthetic melanin (10 mg) was first dissolved in 5 mL of 0.1 N NaOH aqueous solution under vigorous stirring. After dissolving, HCl aqueous solution (0.1 N) was swiftly dropped into the obtained basic melanin solution to adjust the pH to 7.0 under sonication with output power = 10 W for 1 min. A bright black melanin aqueous solution was obtained. The neutralized solution was further centrifuged with a centrifugal-filter (MWCO = 30 kDa) and washed with deionized water and repeated several times to remove the produced NaCl. The aqueous solvent was removed by freeze-drying to obtain 7.5 mg black solid. NH_4OH solution (28 wt %) was added to 5 mL of above black solid aqueous solution (1 mg/mL of water) to adjust the pH of the solution to 9. This mixed solution was added dropwise into NH_2-PEG_{5000}-NH_2 (25 mg) aqueous solution. After vigorous stirring for 12 h, PEG-modified melanin nanoparticle was retrieved by centrifugation with a centrifugal-filter (Amicon centrifugal filter device, MWCO = 30 kDa) and washed with deionized water several times by redispersion/centrifugation processes to remove the unreacted NH_2-PEG_{5000}-NH_2. The aqueous solvent was removed by freeze-drying and obtained M-dots.

Preparation of Cu(I)/M-dots catalyst

The M-dots solubilized in 1 mL of degassed water (1 mg in 1 mL H_2O) and 20 µL of fresh $CuSO_4$ (10 mg/ mL) was successively added. The solution was stirred at 37 °C in 30 min under N_2. Then, 20 eq sodium ascorbate solubilized in degassed water is added dropwise to the solution. After stirring 30 min under N_2, the catalyst was then purified by a centrifugal-filter (MWCO = 30 kDa) and washed with water for three times to obtain Cu(I)/M-dots catalyst and directly used for further applications. The Cu(I) concentration of M-dots was measured by inductively coupled plasma-mass spectrometry (ICP-MS) analysis.

Characterization of M-dots and Cu(I)/M-dots

The hydrodynamic sizes of M-dots and Cu(I)/M-dots were measured by dynamic light scattering (DLS) instrument using a 90 Plus particle size analyzer (Malvern, Zetasizer Nano ZS90). Briefly, 200 μL of M-dots based samples were firstly passed through a 0.22 μm filter and then were filled with a 200 μL clean microcuvette for DLS analysis. We made 6 separate DLS measurements with each measurement consisting of 5 sub-runs with a 10 second duration. Zeta potential was measured using a zeta potential analyzer (Malvern, Zetasizer Nano ZS90). Zeta cells should be rinsed thoroughly and dried using nitrogen before use. The zeta cell was filled with 200 μL of samples by gently depressing the syringe plunger. In order to establish measurement repeatability, each sample was performed for three runs.

The ^1H NMR spectra of M-dots was recorded on a 400 MHz NMR spectrometer (Bruker), using D_2O as solvent. Transmission electron microscopy (TEM) images were recorded on a JEOL 2010 transmission electron microscope at accelerating voltage of 100 kV. The TEM specimens were made by placing a drop of the M-dots and Cu(I)/M-dots aqueous solution on a carbon-coated copper grid, followed by plasma cleaning. Matrix assisted laser desorption/ionization time of flight mass spectrometry (MALDI-TOF-MS) of M-dots was carried out by Stanford Protein and Nucleic Acid Biotechnology Facility, Stanford University. X-ray photoelectron spectra of Cu(I)/M-dots was recorded by using Thermo ESCALAB 250Xi X-ray photoelectron spectrometer. Monochromatic Al Kα X-rays (1486.68 eV) were utilized for excitation. The Cu(I)/M-dots dark powder (obtained by lypophilization) was used for the analysis and kept in a vacuum of 10^{-8} mbar during measurements. For enhancing Cu signal intensities the measurements were recorded at 50 scans. Typically the hydrocarbon C 1s line was taken as an internal standard at 284.8 eV.

Inductively coupled plasma-mass spectrometry (ICP-MS) analysis

Thermo Scientific Xseries 2 Quadrupole was used to measure the concentration of Cu(I) in Cu(I)/M-dots. Standards (0.2 ppm, 0.5 ppm, 1 ppm, 4 ppm and 10 ppm of copper) were prepared in dilute nitric acid (2% w/w) solution and 2% nitric acid solution was used as the blank.

For freshly prepared Cu(I)/M-dots catalyst: 100 μL of detected samples were firstly heated to

evaporate the water solvent and then digested with 0.5 mL of trace metal grade concentrated nitric acid (HNO_3, 70% w/w) under heating until completely dissolved, then the residue was dissolved in 7 mL of dilute nitric acid (2% w/w) for final ICP-MS analysis.

For recycled Cu(I)/M-dots catalyst: after each click reaction, Cu(I)/M-dots were separated immediately from the reaction mixture (30 kDa centrifugal-filter, washed with water for three times) and then stored in water. Recycled samples (100 μL) were prepared firstly and heated to evaporate the water solvent and then digested with 0.5 mL of trace metal grade concentrated nitric acid (HNO_3, 70% w/w) under heating until completely dissolved. Then the residue was dissolved in 7 mL of dilute nitric acid (2% w/w) for final ICP-MS analysis.

The stability of Cu(I)/M-dots catalyst

The stability of Cu(I)/M-dots was studied by incubating those Cu(I)/M-dots in PBS (pH = 7.4) at 37 ^{o}C. Those M-dots were placed in dialysis tube (MWCO 10K) with magnetic stirring, dialysis against 10 ml PBS. At a certain time, dialysate was removed for ICP-MS analysis and replaced with fresh PBS solution. For ICP-MS analysis, 100 μL of the detected sample was firstly heated to evaporate the water solvent and then digested with 0.5 mL of concentrated nitric acid (70% w/w) under heating. After the solvent was evaporated, the residue was then dissolved in 7 mL of dilute nitric acid (2% w/w) for final ICP-MS analysis.

Cell lines

U87MG glioblastoma and NIH3T3 cells were obtained from the American Type Culture Collection (Manassas, VA, USA) and culture media was obtained from Invitrogen Co. (Carlsbad, CA, USA). The cells were cultured in Dulbecco's modified Eagle's medium (DMEM) supplemented with 10% (v/v) fetal bovine serum and 1% (v/v) penicillin at 37°C and 5% CO_2.

Cell viability

In vitro cytotoxicity of Cu(I)/M-dots was determined in NIH3T3 and U87MG cells by the MTT assay. NIH3T3 and U87MG cells were incubated on 96-well plate in DMEM medium containing 10% FBS and 1% penicillin/streptomycin at 37 °C in 5% CO_2 humidified atmosphere for 24 h and 1×10^4 cells were seeded per well. Cu(I)/M-dots and Cu(I)/THPTA (prepared by 1 equivalent of $CuSO_4$ was incubated with 1 equivalent of THPTA for 30 min and sodium ascorbate (30 eq) for 30 min) were added with copper concentrations of 50 μM, 100 μM and 200 μM for 1 h, 12 h, 24 h and 48 h (with n=3 per sample). After 1 h, 12 h, 24 h and 48 h of treatment, cells were washed with PBS and culture medium was replaced with DMEM. Addition of 10 μL of MTT (0.5 mg/mL) solution to each well and incubation for 4 h at 37 °C was followed to produce formazan crystals. Then, the supernatant was removed and the products were lysed with 100 μL of DMSO. The absorbance value was recorded at 490 nm using a microplate reader. The absorbance of the untreated cells was used as a control and its absorbance was as the reference value for calculating 100% cellular viability.

DNA bioconjugation

Oligonucleotide synthesized by Protein and Nucleic Acid (PAN) facility at Stanford. The 3'-position modified oligonucleotide (**S1,** 5 nmol, MW=6317.2) in 20 μL of water was reacted with an excess of NHS ester **V1** (50 eq) in 30 μL of water at room temperature for 12 h. Unreacted **V1** was removed by a centrifugal-filter (3 kDa), and then lyophilized to obtain alkyne-labeled DNA **S2**. A click reaction of **S2** (20 μM) was then performed with Cy5.5-azide (**2f**, 80 μM) mediated by 2 μM Cu(I)/M-dots (containing about 100 μM Cu^+) in 0.1 M PBS at pH 7 for 2h. Unreacted Cy5.5 dye was removed by centrifugal-filter (MWCO = 3 kDa). Then the resulting fluorescent DNA **S3** was separated from the catalyst by centrifugal-filter (MWCO = 10 kDa).

DNA denaturing PAGE Gel for Electrophoresis

The product **S3** was analyzed on a 16% PAGE gel (PAGE gel was prepared by following the standard protocol). The GelRed Nucleic Acid Gel Stain (Biotium) was used to visualize the DNA. Samples were electrophoresed for 3 h at 15 mA, using TBE buffer. The DNA PAGE

gel was imaged under 365 nm UV light (UVP, UV imager) In addition, MALDI-TOF-MS showed the expected molecular weight for the corresponding cyclo-adduct (MW=7299.9).

Cell Labeling with CuAAC probes for fluorescence microscopy imaging.

U87MG cells (1×10^5) were suspended in 500 μL of DMEM seeded in 12-well tissue culture plates and incubated at 37°C for overnight. Then, U87MG cells were incubated first with RGD-alkyne (10 μM) for 30 min at 37°C. After several washing steps to remove unbound alkyne, the Cy5.5-azide (**2f**, 30 μM) and 4 μM Cu(I)/M-dots (containing about 200 μM Cu^+) were added to the cell culture medium for incubation for 60 min at 37°C. After 1h click reaction for labeling live cells, the U87MG cells were washed three times with PBS and then fixed with fresh 4% paraformaldehyde (PFA) for 5 min at room temperature. Cell nuclei were stained by adding 4',6-diamidino-2-phenylindole (DAPI). The cells were then imaged using fluorescence microscopy (Axiovert 200M fluorescence microscope).

Chemical synthesis and characterization

General procedure for the two-componets [3+2] cyclo-addition reaction.

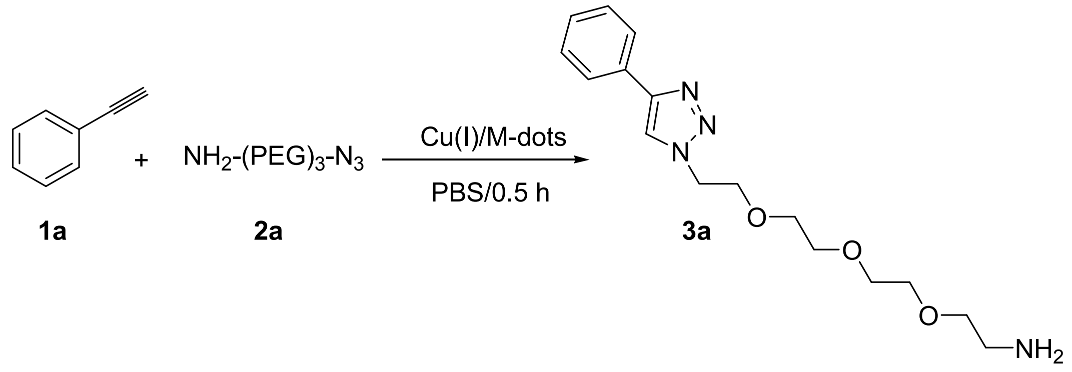

Synthesis of **3a**: To a solution of **1a** (10.2 mg, 0.1 mmol) and compound **2a** (26.2 mg, 0.12 mmol, 1.2 equiv) in PBS (1 mL) at room temperature was added 2 nmol Cu(I)/M-dots (containing 0.1% mol Cu^+). The reaction mixture was stirred at this temperature for 0.5 h. The catalyst was separated by a centrifugal-filter (MWCO = 30 kDa), and the residue solvent was lyophilized to obtain the crude product. The crude product was purified by flash chromatography affording the desired product **3a** (30.0 mg, 94% yield). ^{1}H NMR (400 MHz,

D_2O):δ = 8.16 (s, 1H), 7.63 (s, 2H), 7.37-7.31 (m, 3H), 4.49 (s, 2H), 3.84 (s, 2H), 3.56-3.34 (m, 12H), 2.91 (s, 2H); ^{13}C NMR (101 MHz, D_2O) δ = 147.20, 129.25, 129.17, 128.79, 69.60, 69.43, 69.35, 69.20, 68.47, 66.14, 50.12, 38.88; HRMS (ESI) Calcd. for: $C_{16}H_{25}N_4O_3^+$ ($[M+H]^+$): 321.1921, found: 321.1917.

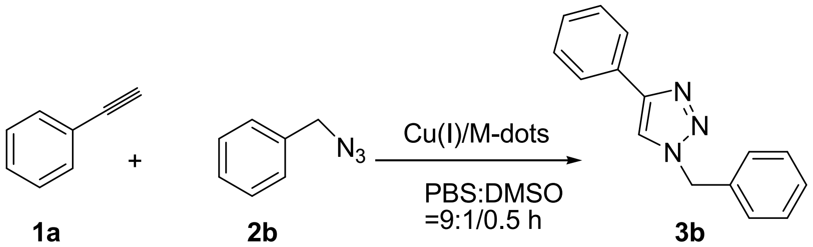

1a 2b 3b

Synthesis of **3b**: To a solution of **1a** (10.2 mg, 0.1 mmol) and compound **2b** (15.9 mg, 0.12 mmol, 1.2 equiv) in PBS/DMSO (9:1) at room temperature was added 2 nmol Cu(I)/M-dots (containing 0.1% mol Cu^+). The reaction mixture was stirred at this temperature for 0.5 h. The catalyst was separated by a centrifugal-filter (MWCO = 30 kDa), and the residue solvent was lyophilized to obtain the crude product. The crude product was purified by flash chromatography affording the desired product **3b** (21.1 mg, 90% yield). ^1H NMR (400 MHz, MeOD):δ = 8.30 (s, 1H), 7.79 (s, 2H), 7.38 (m, 7H), 5.63 (s, 2H); ^{13}C NMR (101 MHz, MeOD) δ = 147.80, 135.38, 130.20, 128.63, 128.53, 128.18, 127.94, 127.65, 125.24, 120.79, 109.99, 53.62; HRMS (ESI) Calcd. for: $C_{15}H_{14}N_3^+$ ($[M+H]^+$): 236.1182, found: 236.1185.

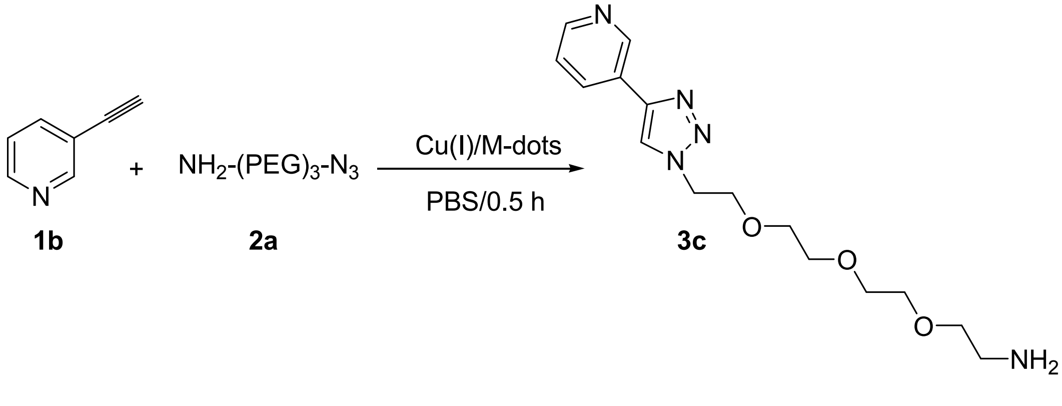

1b 2a 3c

Synthesis of **3c**: To a solution of **1b** (10.3 mg, 0.1 mmol) and compound **2a** (26.2 mg, 0.12 mmol, 1.2 equiv) in PBS at room temperature was added 2 nmol Cu(I)/M-dots (containing 0.1% mol Cu^+). The reaction mixture was stirred at this temperature for 0.5 h. The catalyst was

separated by a centrifugal-filter (MWCO = 30 kDa), and the residue solvent was lyophilized to obtain the crude product. The crude product was purified by flash chromatography affording the desired product **3c** (28.9 mg, 90% yield). ^{1}H NMR (400 MHz, D_2O):δ = 8.96 (s, 1H), 8.70 (s, 1H), 8.33-7.93 (m, 3H), 4.70 (s, 2H), 4.02-3.97 (m, 2H), 3.36-3.61 (m, 9H), 3.11 (s, 2H); ^{13}C NMR (101 MHz, D_2O) δ = 123.91, 70.03, 69.93, 69.91, 69.78, 68.78, 66.40, 50.31, 39.19; HRMS (ESI) Calcd. for: $C_{15}H_{24}N_5O_3^+$ ([M+H]$^+$): 322.1874, found: 322.1871.

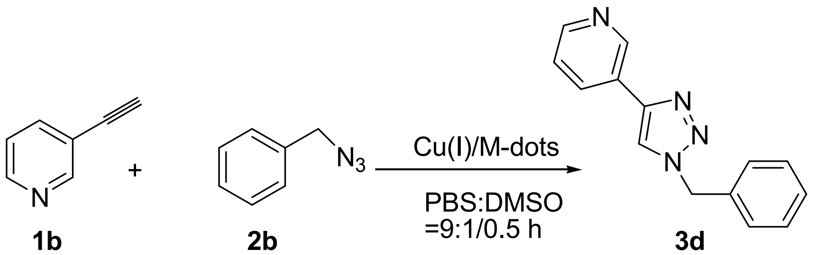

1b **2b** **3d**

Synthesis of **3d**: To a solution of **1b** (10.3 mg, 0.1 mmol) and compound **2b** (15.9 mg, 0.12 mmol, 1.2 equiv) in PBS/DMSO (9:1) at room temperature was added 2 nmol Cu(I)/M-dots (containing 0.1% mol Cu^+). The reaction mixture was stirred at this temperature for 0.5 h. The catalyst was separated by a centrifugal-filter (MWCO = 30 kDa), and the residue solvent was lyophilized to obtain the crude product. The crude product was purified by flash chromatography affording the desired product **3d** (21.2 mg, 90% yield). ^{1}H NMR (400 MHz, MeOD):δ = 9.22 (s, 1H), 8.81-8.62 (m, 3H), 7.96 (s, 1H), 7.40 (s, 4H), 5.69 (s, 2H); ^{13}C NMR (101 MHz, MeOD) δ = 141.74, 141.34, 140.64, 139.33, 134.97, 130.53, 128.72, 128.39, 127.89, 126.99, 123.20; HRMS (ESI) Calcd. for: $C_{14}H_{13}N_4^+$ ([M+H]$^+$): 237.1135, found: 237.1135.

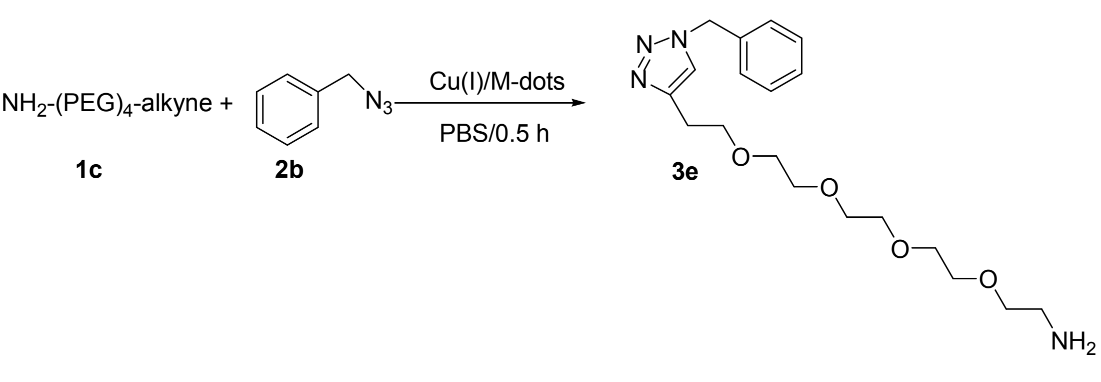

1c **2b** **3e**

Synthesis of **3e**: To a solution of **1c** (24.5 mg, 0.1 mmol) and compound **2b** (15.9 mg, 0.12

mmol, 1.2 equiv) in PBS at room temperature was added 2 nmol Cu(I)/M-dots (containing 0.1% mol Cu^+). The reaction mixture was stirred at this temperature for 0.5 h. The catalyst was separated by a centrifugal-filter (MWCO = 30 kDa), and the residue solvent was lyophilized to obtain the crude product. The crude product was purified by flash chromatography affording the desired product **3e** (35.9 mg, 95% yield). ^{1}H NMR (400 MHz, D_2O):δ = 7.96 (s, 1H), 7.39-7.32 (m, 5H), 5.59 (s, 2H), 4.62 (s, 2H), 3.70-3.57 (m, 20H), 3.31 (d, J = 4.0 Hz, 2H), 3.07-3.04 (m, 2H); ^{13}C NMR (101 MHz, D_2O) δ = 144.67, 135.32, 128.62, 128.23, 127.74, 123.50, 70.01, 69.81, 69.76, 69.73, 69.40, 69.33, 66.36, 63.40, 53.54, 38.99; HRMS (ESI) Calcd. for: $C_{19}H_{31}N_4O_4^+$ ([M+H]$^+$): 379.2340, found: 379.2336.

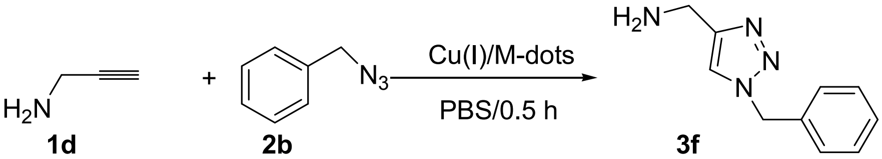

1d **2b** **3f**

Synthesis of **3f**: To a solution of **1d** (5.5 mg, 0.1 mmol) and compound **2b** (15.9 mg, 0.12 mmol, 1.2 equiv) in PBS at room temperature was added 2 nmol Cu(I)/M-dots (containing 0.1% mol Cu^+). The reaction mixture was stirred at this temperature for 0.5 h. The catalyst was separated by a centrifugal-filter (MWCO = 30 kDa), and the residue solvent was lyophilized to obtain the crude product. The crude product was purified by flash chromatography affording the desired product **3f** (16.9 mg, 90% yield). ^{1}H NMR (400 MHz, MeOD): δ = 8.04 (s, 1H), 7.35 (s, 5H), 5.61 (s, 2H), 4.42 (s, 2H); ^{13}C NMR (101 MHz, MeOD) δ = 140.15, 135.13, 128.63, 128.29, 127.82, 123.97, 53.63, 34.00; ESI-MS Calcd. for: $C_{10}H_{13}N_4^+$ ([M+H]$^+$): 189.1135, found: 189.1132.

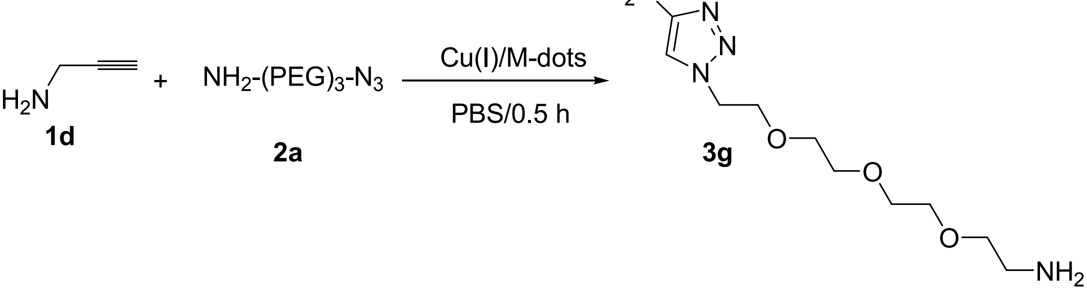

1d **2a** **3g**

Synthesis of **3g**: To a solution of **1d** (5.5 mg, 0.1 mmol) and compound **2a** (26.16 mg, 0.12 mmol, 1.2 equiv) in PBS at room temperature was added 2 nmol Cu(I)/M-dots (containing 0.1%

mol Cu^{+}). The reaction mixture was stirred at this temperature for 0.5 h. The catalyst was separated by a centrifugal-filter (MWCO = 30 kDa), and the residue solvent was lyophilized to obtain the crude product. The crude product was purified by HPLC affording the desired product **3g** (24.6 mg, 95% yield). ^{1}H NMR (400 MHz, MeOD): δ = 8.10 (s, 1H), 4.61 (s, 2H), 4.24 (s, 2H), 3.90 (s, 2H), 3.79-3.61 (m, 10H), 3.12 (s, 2H); ^{13}C NMR (101 MHz, MeOD) δ = 124.65, 76.08, 70.05, 69.94, 69.86, 69.81, 68.91, 66.43, 50.02, 39.21, 34.03, 28.48; ESI-MS Calcd. for: $C_{10}H_{22}N_{5}O_{3}^{+}$ ([M+H]$^{+}$): 260.1717, found: 260.1714.

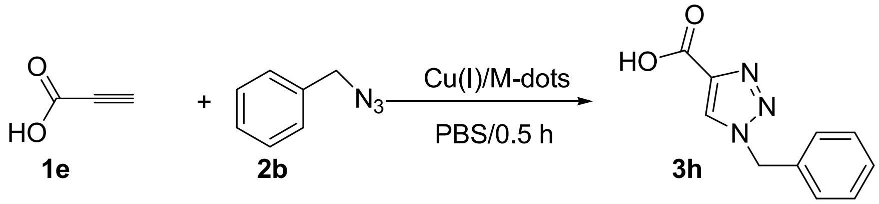

Synthesis of **3h**: To a solution of compound **1e** (7.0 mg, 0.1 mmol) and compound **2b** (15.9 mg, 0.12 mmol, 1.2 equiv) in PBS at room temperature was added 2 nmol Cu(I)/M-dots (containing 0.1% mol Cu^{+}). The reaction mixture was stirred at this temperature for 0.5 h. The catalyst was separated by a centrifugal-filter (MWCO = 30 kDa), and the residue solvent was lyophilized to obtain the crude product. The crude product was purified by flash chromatography affording the desired product **3h** (18.2 mg, 90% yield). ^{1}H NMR (400 MHz, MeOD):δ = 8.48 (br, 1H), 7.36 (s, 5H), 5.65 (s, 2H); ^{13}C NMR (101 MHz, MeOD) δ = 161.86, 1400.03, 134.88, 128.68, 128.34, 128.08, 127.81, 53.70; ESI-MS Calcd. for: $C_{10}H_{10}N_{3}O_{2}^{+}$ ([M+H]$^{+}$): 204.0768, found: 204.0765.

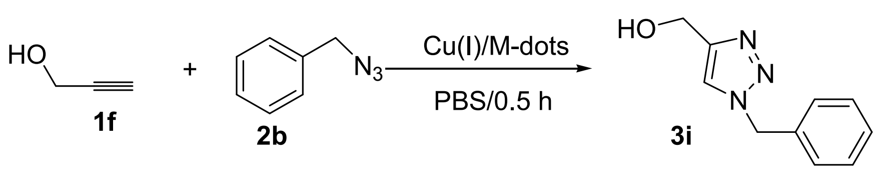

Synthesis of **3i**: To a solution of compound **1f** (5.6 mg, 0.1 mmol) and compound **2b** (15.9 mg, 0.12 mmol, 1.2 equiv) in PBS at room temperature was added 2 nmol Cu(I)/M-dots (containing 0.1% mol Cu^{+}). The reaction mixture was stirred at this temperature for 0.5 h. The catalyst was separated by a centrifugal-filter (MWCO = 30 kDa), and the residue solvent was lyophilized to obtain the crude product. The crude product was purified by flash

chromatography affording the desired product **3i** (17.4 mg, 92% yield). ^{1}H NMR (400 MHz, MeOD):δ = 7.96 (s, 1H), 7.38-7.31 (m, 5H), 5.58 (s, 2H), 4.68 (s, 2H); ^{13}C NMR (101 MHz, MeOD) δ = 135.15, 128.66, 128.28, 127.76, 54.86, 53.74; ESI-MS Calcd. for: $C_{10}H_{12}N_3O^+$ ($[M+H]^+$): 190.0975, found: 190.0978.

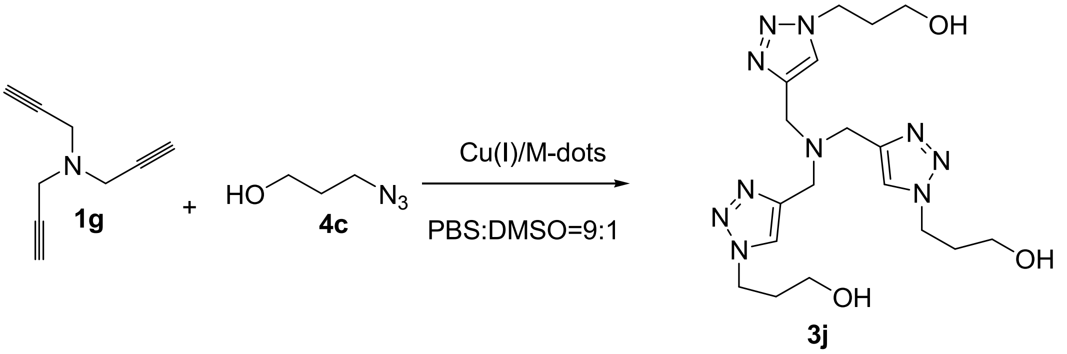

Synthesis of **3j**: To a solution of compound **1g** (13.1 mg, 0.1 mmol) and compound **2c** (36.4 mg, 0.36 mmol, 3.6 equiv) in PBS/DMSO (9:1) at room temperature was added 6 nmol Cu(I)/M-dots (containing 0.3% mol Cu^+). The reaction mixture was stirred at this temperature for 0.5 h. The catalyst was separated by a centrifugal-filter (MWCO = 30 kDa), and the residue solvent was lyophilized to obtain the crude product. The crude product was purified by flash chromatography affording the desired product **3j** (38.2 mg, 90% yield). ^{1}H NMR (400 MHz, MeOD):δ = 7.98 (s, 3H), 4.52-4.49 (m, 6H), 3.74 (s, 6H), 3.58-3.55 (m, 6H), 2.14-2.07 (m, 6H); ^{13}C NMR (101 MHz, MeOD) δ = 143.78, 124.26, 57.89, 32.54; ESI-MS Calcd. for: $C_{18}H_{31}N_{10}O_3^+$ ($[M+H]^+$): 435.2575, found: 435.2575.

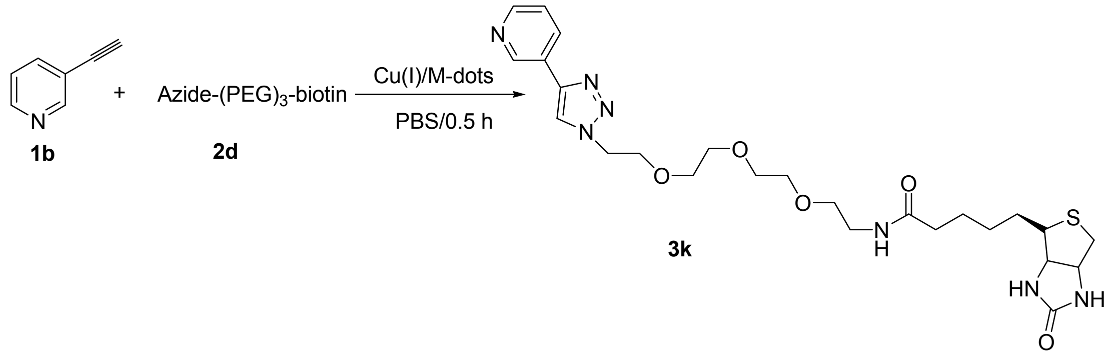

Synthesis of **3k**: To a solution of **1b** (5.2 mg, 0.05 mmol) and compound **2d** (26.64 mg, 0.06 mmol, 1.2 equiv) in PBS at room temperature was added 2 nmol Cu(I)/M-dots (containing 0.1%

mol Cu^+). The reaction mixture was stirred at this temperature for 0.5 h. The catalyst was separated by a centrifugal-filter (MWCO = 30 kDa), and the residue solvent was lyophilized to obtain the crude product. The crude product was purified by HPLC affording the desired product **3k** (28.9 mg, 90% yield). ^1H NMR (400 MHz, MeOD):δ = 8.37 (s, 1H), 7.83-7.81 (d *J* = 8.0 Hz, 2H), 7.45-7.33 (m, 3H), 4.65-4.62 (m, 2H), 4.48-4.45 (m, 1H), 4.29-4.26 (m, 1H), 3.96-3.93 (m, 2H), 3.63-3.43 (m, 10H), 3.19-3.14 (m, 1H), 2.92-2.88 (m, 1H), 2.70-2.67 (m, 1H), 2.19-2.15 (m, 2H), 1.73-1.57 (m, 4H), 1.54-1.38 (m, 2H),; ^{13}C NMR (101 MHz, MeOD) δ = 174.66, 164.69, 130.28, 128.62, 127.97, 125.25, 121.77, 70.10, 70.03, 69.78, 69.09, 68.89, 61.93, 60.20, 55.56, 50.16, 39.61, 38.86, 35.27, 28.32, 28.06, 25.40; ESI-MS Calcd. for: $C_{25}H_{38}N_7O_5S^+$ ($[M+H]^+$): 548.2650, found: 548.2654.

RGDyK + NHS-(PEG)$_3$-alkyne (**V1**) —DIPEA, DMF→ RGDyK-(PEG)$_3$-alkyne (**1h**)

Synthesis of **1h** To a solution of RGDyK (3.10 mg, 5 μmol) and **V1** (2.34 mg, 7.5 μmol, 1.5 equiv) in DMF at room temperature was added DIPEA (0.1 equiv). The reaction mixture was stirring at this temperature for 4 h. The crude product was purified by HPLC. Lyophilization of the purified material gave 3.19 mg (78%) of **1h**. ^1H NMR (400 MHz, D_2O):δ = 8.67 (d *J* = 8.0 Hz, 1H), 8.53-8.49 (m, 1H), 7.98-7.95 (m, 1H),7.27-7.12 (m, 7H), 4.52-4.48 (m, 2H), 4.25-4.21 (m, 2H), 4.12-4.10 (m, 3H), 4.06 (s, 2H), 3.76-3.72 (m, 2H), 3.68-3.65 (m, 3H), 3.63-3.61 (m, 3H), 3.58-3.55 (m, 12H), 3.39 (s, 1H), 3.35 (s, 1H), 3.11-2.93 (m, 8H), 2.87-2.74 (m, 5H), 2.62-2.53 (m, 3H), 2.41-2.38 (m, 3H), 1.78-1.69 (m, 2H), 1.58-1.48 (m, 3H), 1.44-1.33 (m, 4H), 1.27-1.20 (m, 3H), 0.98 (d *J* = 8.0 Hz, 1H), 0.87-0.79 (m, 3H), ESI-MS Calcd. for: $C_{37}H_{56}N_9O_{12}^+$ ($[M+H]^+$):818.4, found: 819.2.

AE105 + NHS-(PEG)$_3$-alkyne (**V1**) —DIPEA, DMF→ AE105-(PEG)$_3$-alkyne (**1i**)

Synthesis of **1i** To a solution of AE105 (1.45 mg, 1 μmol) and **V1** (0.47 mg, 1.5 μmol, 1.5 equiv) in DMF at room temperature was added DIPEA (0.1 equiv). The reaction mixture was stirring at this temperature for 4 h. The crude product was purified by HPLC. Lyophilization

of the purified material gave 1.28 mg (78%) of **1h**. ESI-MS Calcd. for: $C_{80}H_{116}N_{17}O_{21}^+$ ($[M+H]^+$):1650.8, found: 1651.2.

RGDyK + NHS-$(PEG)_3$-azide (**V2**) —DIPEA / DMF→ RGDyK-$(PEG)_3$-azide (**2e**)

Synthesis of **2e** To a solution of RGDyK (3.10 mg, 5 µmol) and **V2** (2.58 mg, 7.5 µmol, 1.5 equiv) in DMF at room temperature was added DIPEA (0.1 equiv). The reaction mixture was stirring at this temperature for 4 h. The crude product was purified by HPLC. Lyophilization of the purified material gave 3.02 mg (71%) of **2e**. ^1H NMR (400 MHz, D_2O):δ = 8.67 (d J = 4.0 Hz, 1H), 8.53-8.49 (m, 1H), 7.98-7.95 (m, 1H),7.27-7.12 (m, 7H), 4.52-4.48 (m, 2H), 4.25-4.21 (m, 2H), 4.12-4.10 (m, 3H), 4.06 (s, 2H), 3.76-3.72 (m, 2H), 3.68-3.65 (m, 3H), 3.63-3.61 (m, 3H), 3.58-3.55 (m, 12H), 3.39 (s, 1H), 3.35 (s, 1H), 3.11-2.93 (m, 8H), 2.87-2.74 (m, 5H), 2.62-2.53 (m, 3H), 2.41-2.38 (m, 3H), 1.78-1.69 (m, 2H), 1.58-1.48 (m, 3H), 1.44-1.33 (m, 4H), 1.27-1.20 (m, 3H), 0.98 (d J = 8.0 Hz, 1H), 0.87-0.79 (m, 3H). ESI-MS Calcd. for: $C_{36}H_{57}N_{12}O_{12}^+$ ($[M+H]^+$): 849.4, found: 849.1.

Cy5.5-NHS + NH_2-$(PEG)_3$-N_3 (**2a**) —DIPEA / DMF→ Cy5.5-$(PEG)_3$-azide (**2f**)

Synthesis of **2f** To a solution of Cy5.5 NHS ester (0.70 mg, 1 µmol) and **2a** (0.26 mg, 1.2 µmol, 1.2 equiv) in DMF at room temperature was added DIPEA (0.1 equiv). The reaction mixture was stirring at this temperature for 4 h. The crude product was purified by HPLC. Lyophilization of the purified material gave 0.42 mg (50%) of **2f**. ESI-MS Calcd. for: $C_{48}H_{60}N_6O_4^+$ ($[M+H]^+$): 784.4, found: 784.2.

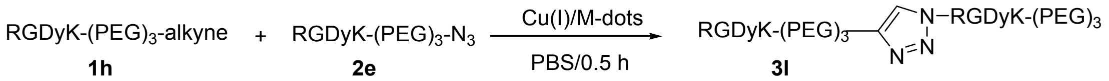

Synthesis of **3l**: To a solution of **1h** (1.23 mg, 1.5 µmol) and compound **2e** (1.87 mg, 2.2 µmol, 1.5 equiv) in PBS at room temperature was added 0.03 nmol Cu(I)/M-dots (containing 0.1% mol Cu^+). The reaction mixture was stirred at this temperature for 0.5 h. The catalyst was separated by a centrifugal-filter (MWCO = 30 kDa), and the residue solvent was lyophilized to obtain the crude product. The crude product was purified by HPLC affording the desired

product **3l** (1.87 mg, 75% yield); ESI-MS Calcd. for: $C_{73}H_{112}N_{21}O_{24}^+$ ([M+H]$^+$): 1666.8, found:1666.5.

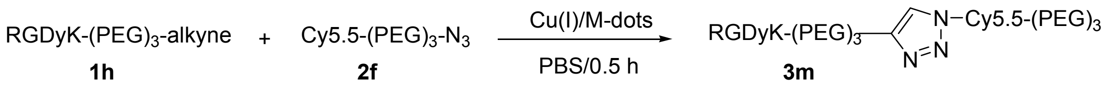

Synthesis of **3m**: To a solution of **1h** (1.23 mg, 1.5 μmol) and compound **2f** (1.73 mg, 2.2 μmol, 1.5 equiv) in PBS at room temperature was added 0.03 nmol Cu(I)/M-dots (containing 0.1% mol Cu^+). The reaction mixture was stirred at this temperature for 0.5 h. The catalyst was separated by a centrifugal-filter (MWCO = 30 kDa), and the residue solvent was lyophilized to obtain the crude product. The crude product was purified by HPLC affording the desired product **3m** (1.87 mg, 78% yield); ESI-MS Calcd. for: $C_{85}H_{115}N_{15}O_{16}^+$ ([M+H]$^+$): 1601.8, found:1602.5.

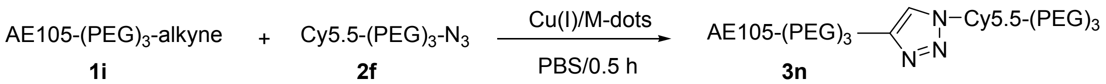

Synthesis of **3n**: To a solution of **1i** (1.65 mg, 1.0 μmol) and compound **2f** (1.17 mg, 1.5 μmol, 1.5 equiv) in PBS at room temperature was added 0.02 nmol Cu(I)/M-dots (containing 0.1% mol Cu^+). The reaction mixture was stirred at this temperature for 0.5 h. The catalyst was separated by a centrifugal-filter (MWCO = 30 kDa), and the residue solvent was lyophilized to obtain the crude product. The crude product was purified by HPLC affording the desired product **3n** (1.86 mg, 75% yield); MALDI-TOF-MS: $C_{128}H_{175}N_{23}O_{25}^+$ ([M+H]$^+$): 2434.3, found:2435.1.

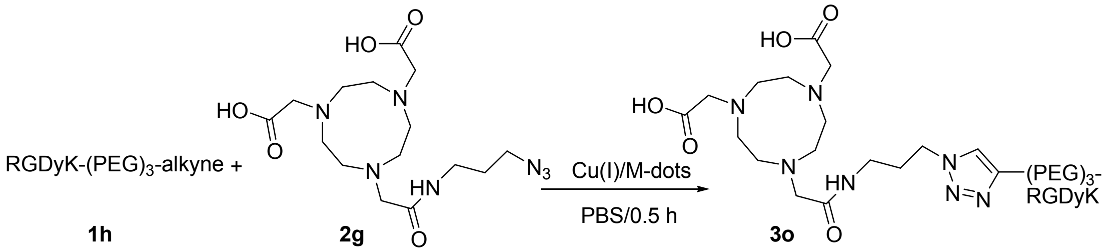

Synthesis of **3o**: To a solution of **1h** (0.82 mg, 1.0 μmol) and compound **2g** (0.575 mg, 1.5 μmol, 1.5 equiv) in PBS at room temperature was added 0.02 nmol Cu(I)/M-dots (containing 0.1% mol Cu^+). The reaction mixture was stirred at this temperature for 0.5 h. The catalyst was separated by a centrifugal-filter (MWCO = 30 kDa), and the residue solvent was lyophilized to obtain the crude product. The crude product was purified by HPLC affording

the desired product **3o** (1.04 mg, 84% yield); ESI-MS Calcd. for: $C_{52}H_{83}N_{16}O_{17}^+$ ([M+H]$^+$): 1203.6, found:1203.3.

Synthesis of **AE105:** Peptide AE105 (Ac-Lys-Gly-Asp-Cha-Phe-(D)Ser-(D)Arg-Tyr-Leu- -Trp-Ser-NH_2) was synthesized on Tentagel S RAM resin using traditional Fmoc solid-phase peptide chemistry. After deprotection and cleavage from the resin using 93% TFA, 5% Tips, and 2% H_2O for 2 h, the peptide was precipitated in cold Et_2O and washed with Et_2O three times. The dried peptide was purified by prep-HPLC. MS Calcd for: $C_{70}H_{102}N_{17}O_{17}^+$ ([M+H]$^+$): 1452.8, found: MALDI-MS: m/z 1452.3.

General Procedure for the Three-Componet [3+2] Cyclo-addition Reaction.

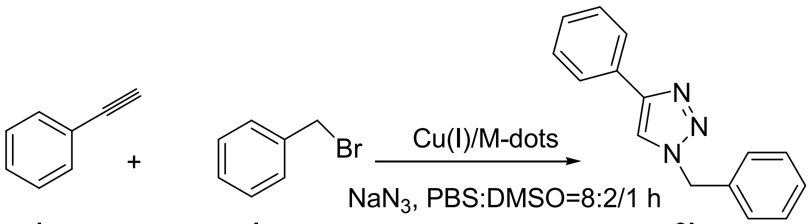

1a **4a** **3b**

Synthesis of **3b**: To a solution of **1a** (10.2 mg, 0.1 mmol), Compound **4a** (20.4 mg, 0.12 mmol, 1.2 equiv) and sodium azide (7.8 mg, 0.12 mmol) in PBS/DMSO (8:2) at room temperature was added 2 nmol Cu(I)/M-dots (containing 0.1% mol Cu$^+$). The reaction mixture was stirred at this temperature for 1 h. The catalyst was separated by a centrifugal-filter (MWCO = 30 kDa), and the residue solvent was lyophilized to obtain the crude product. The crude product was purified by flash chromatography affording the desired product **3b** (20.6 mg, 88% yield).

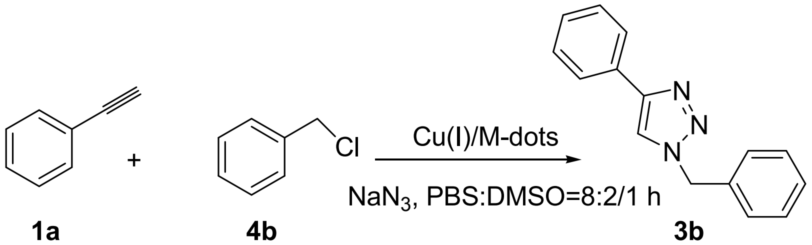

1a **4b** **3b**

Synthesis of **3b**: To a solution of **1a** (10.2 mg, 0.1 mmol), Compound **4b** (15.1 mg, 0.12 mmol, 1.2 equiv) and sodium azide (7.8 mg, 0.12 mmol) in PBS/DMSO (8:2) at room temperature

was added 2 nmol Cu(I)/M-dots (containing 0.1% mol Cu^+). The reaction mixture was stirred at this temperature for 1 h. The catalyst was separated by a centrifugal-filter (MWCO = 30 kDa), and the residue solvent was lyophilized to obtain the crude product. The crude product was purified by flash chromatography affording the desired product **3b** (19.9 mg, 85% yield).

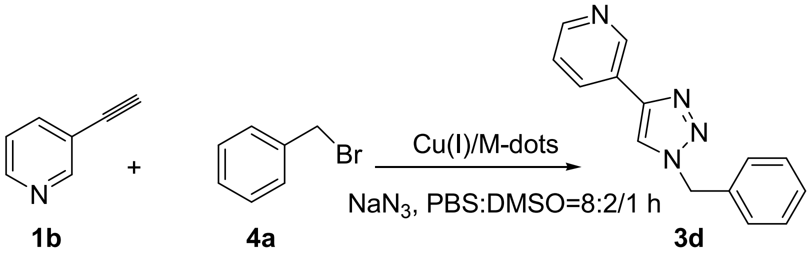

Synthesis of **3d**: To a solution of **1b** (10.3 mg, 0.1 mmol), Compound **4a** (20.4 mg, 0.12 mmol, 1.2 equiv) and sodium azide (7.8 mg, 0.12 mmol) in PBS/DMSO (8:2) at room temperature was added 2 nmol Cu(I)/M-dots (containing 0.1% mol Cu^+). The reaction mixture was stirred at this temperature for 1 h. The catalyst was separated by a centrifugal-filter (MWCO = 30 kDa), and the residue solvent was lyophilized to obtain the crude product. The crude product was purified by flash chromatography affording the desired product **3d** (18.4 mg, 78% yield).

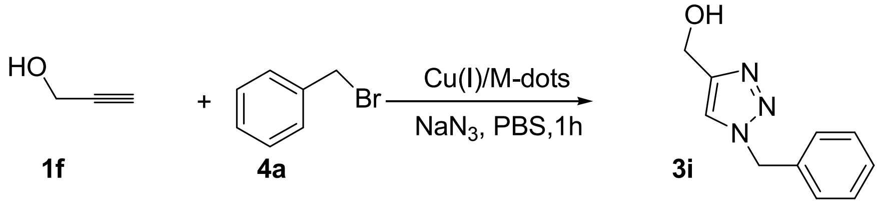

Synthesis of **3i**: To a solution of **1f** (0.56 mg, 0.1 mmol), Compound **4a** (20.4 mg, 0.12 mmol, 1.2 equiv) and sodium azide (7.8 mg, 0.12 mmol) in PBS at room temperature was added 2 nmol Cu(I)/M-dots (containing 0.1% mol Cu^+). The reaction mixture was stirred at this temperature for 1 h. The catalyst was separated by a centrifugal-filter (MWCO = 30 kDa), and the residue solvent was lyophilized to obtain the crude product. The crude product was purified by flash chromatography affording the desired product **3i** (17.1 mg, 90% yield).

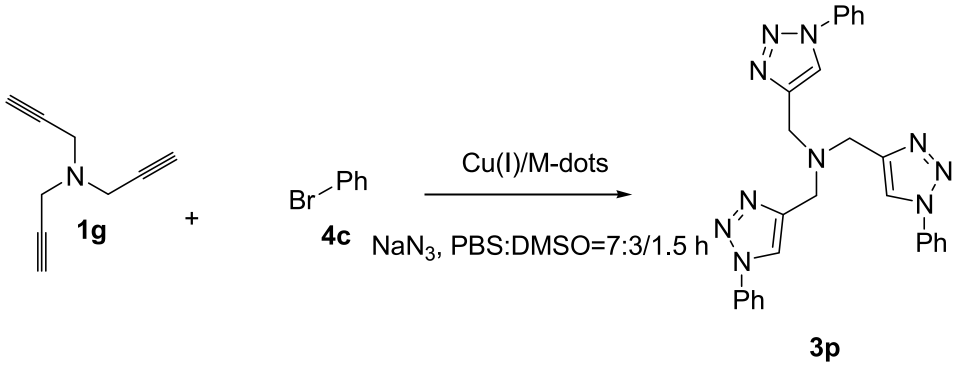

Synthesis of **3p**: To a solution of compound **1g** (13.1 mg, 0.1 mmol), compound **4c** (55.8 mg, 0.36 mmol, 3.6 equiv) and sodium azide (23.4 mg, 0.36 mmol) in PBS/DMSO (7:3) at room temperature was added 6 nmol Cu(I)/M-dots (containing 0.3% mol Cu^+). The reaction mixture was stirred at this temperature for 1.5 h. The catalyst was separated by a centrifugal-filter (MWCO = 30 kDa), and the residue solvent was lyophilized to obtain the crude product. The crude product was purified by flash chromatography affording the desired product **3p** (32.7 mg, 67% yield). ^{1}H NMR (400 MHz, $CDCl_3$):δ = 8.17 (s, 3H), 7.37-7.26 (m, 15H), 5.53 (s, 6H), 4.14 (s, 6H); ^{13}C NMR (101 MHz, $CDCl_3$) δ = 134.05, 129.19, 128.92, 128.92, 128.09, 54.49, 46.17 ^{1}H NMR (400 MHz, MeOD):δ = 7.98 (s, 3H), 4.52-4.49 (m, 6H), 3.74 (s, 6H), 3.58-3.55 (m, 6H), 2.14-2.07 (m, 6H); ^{13}C NMR (101 MHz, MeOD) δ = 143.78, 124.26, 57.89, 32.54; ESI-MS Calcd for: $C_{27}H_{25}N_{10}^+$ ([M+H]$^+$): 489.2258, found: 489.2254.

In vitro Kinetic study

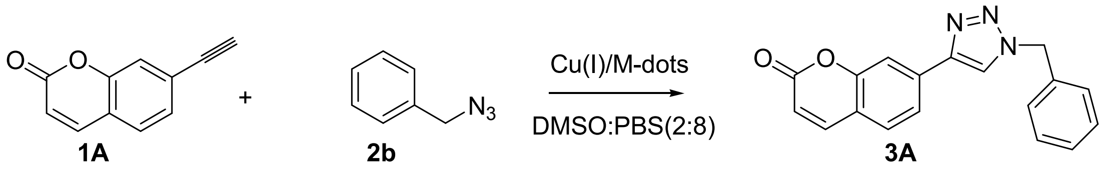

General conditions: 50 μM 1A, 25 μM benzyl azide and Cu(I)/M-dots in 10 ml PBS:DMSO (8:2). The reaction mixture was purified by HPLC to obtain the desired product 3A. ESI-MS Calcd. for: $C_{18}H_{14}N_3O_2^+$ ([M+H]$^+$): 304.1008, found: 304.1015. Coumarin fluorescence was recorded on a Tecan SAFIRE microplate reader at 2-min intervals for 30 min with excitation at 320 nm and emission detected at 400 nm.

The free radical quenching ability of M-dots in CuAAC conditions.

The ascorbate-driven, metal-induced hydroxyl radical production was monitored by HPLC

using the hydroxyl radical scavenging compound 3-CCA (sigma-aldrich). The hydroxyl radical reaction was carried out with or without M-dots (100 μM) in the presence of 100 μM 3-CCA and ascorbate (5 mM) after 12h.

Recycle study for the Two-Components [3+2] cyclo-addition reaction.

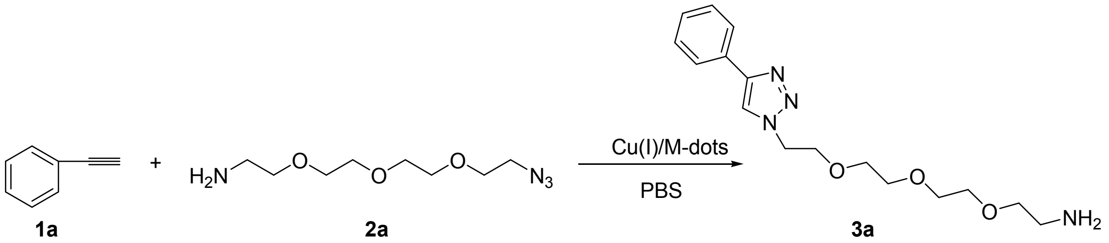

General conditions: To a solution of **1a** (10.2 mg, 0.1 mmol) and compound **2a** (26.2 mg, 0.12 mmol, 1.2 equiv) in PBS at room temperature was added 2 nmol Cu(I)/M-dots (containing 0.1% mol Cu^{+}). The reaction mixture was stirred at this temperature for 0.5 h. The catalyst was separated by a centrifugal-filter (MWCO = 30 kDa), and washed with water for three times. Then the recycle catalyst was reused directly in the next reaction.

Recycle study for the Three-Components [3+2] cyclo-addition reaction.

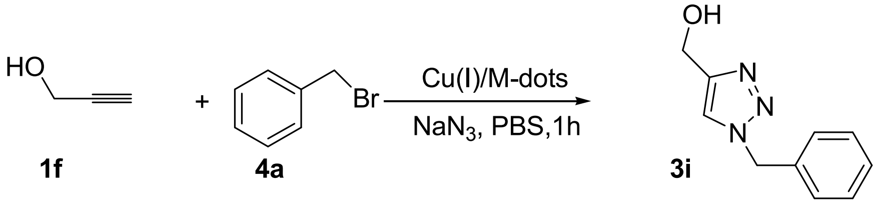

General conditions: To a solution of **1f** (5.6 mg, 0.1 mmol), Compound **4a** (20.4 mg, 0.12 mmol, 1.2 equiv) and sodium azide (7.8 mg, 0.12 mmol) in PBS at room temperature was added 2 nmol Cu(I)/M-dots (containing 0.1% mol Cu^{+}). The reaction mixture was stirred at this temperature for 1.5 h. The catalyst was separated by a centrifugal-filter (MWCO = 30 kDa), and washed with water for three times. Then the recycle catalyst was reused directly in the next reaction.

Radiochemistry

The ^{64}**Cu-3o** was radiosynthesized as previously described. Probe precursor **3o** (20 μg) was incubated with 1 mCi $^{64}CuCl_2$ at room temperature in 0.1 M sodium acetate buffer (pH 5.5) for 30 min and was analyzed using RP-HPLC with a C18 column.

NMR Spectra

(a). ^1H NMR and ^{13}C NMR for 3a

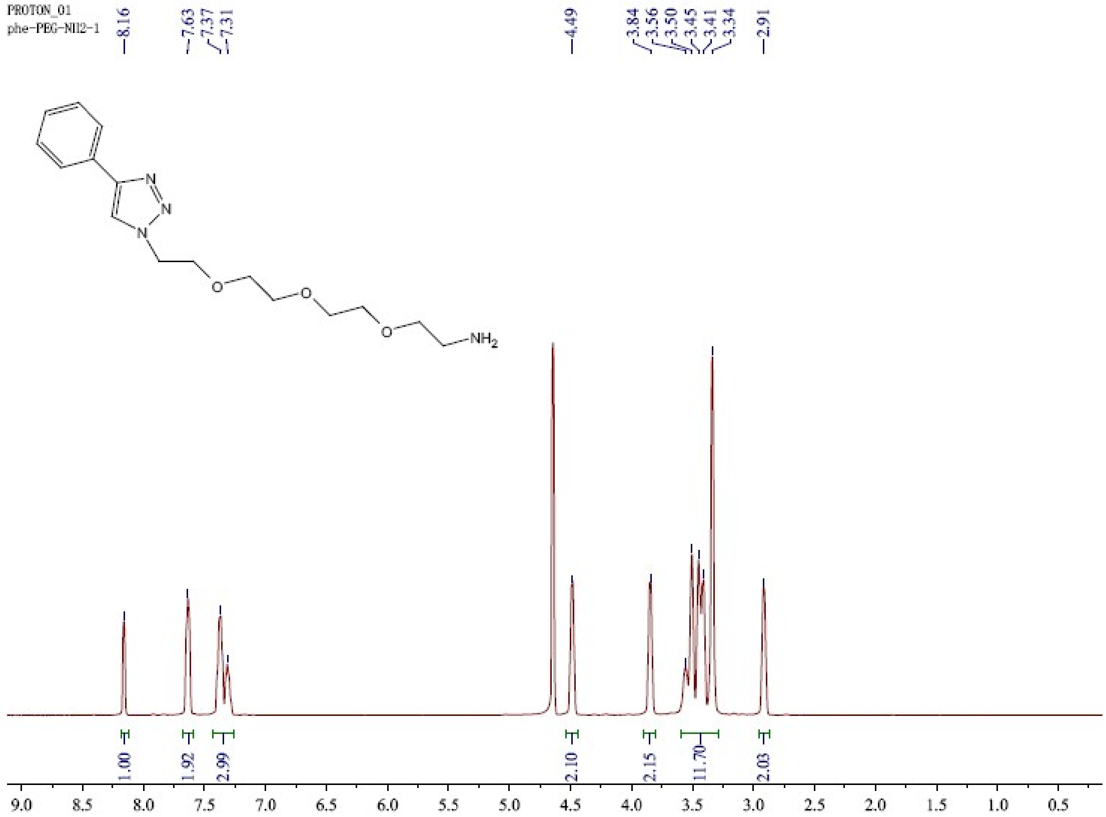

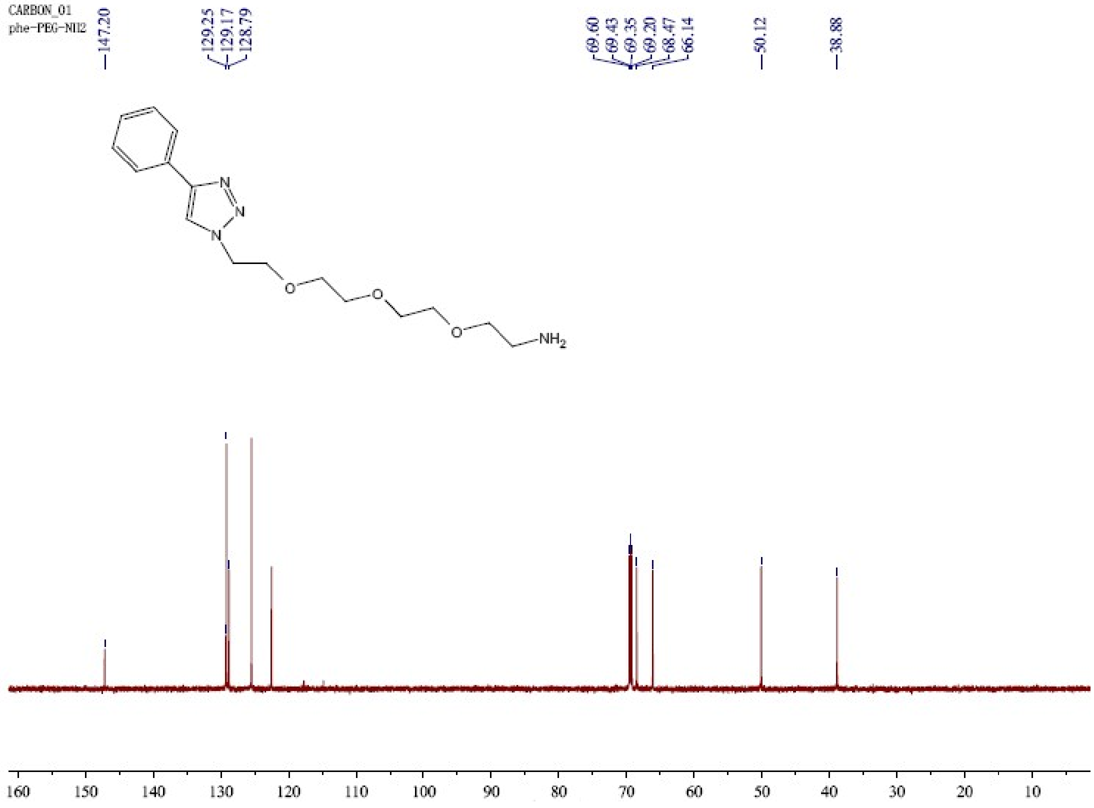

(b). ^{1}H NMR and ^{13}C NMR for 3b

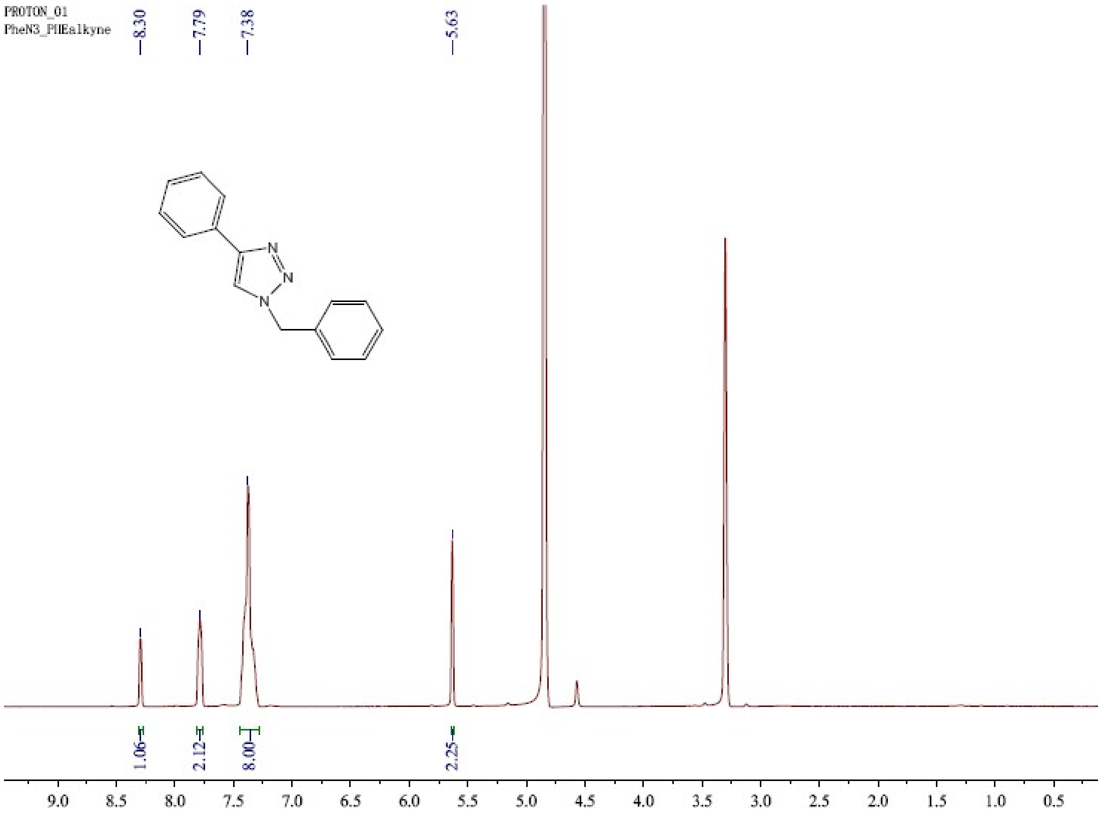

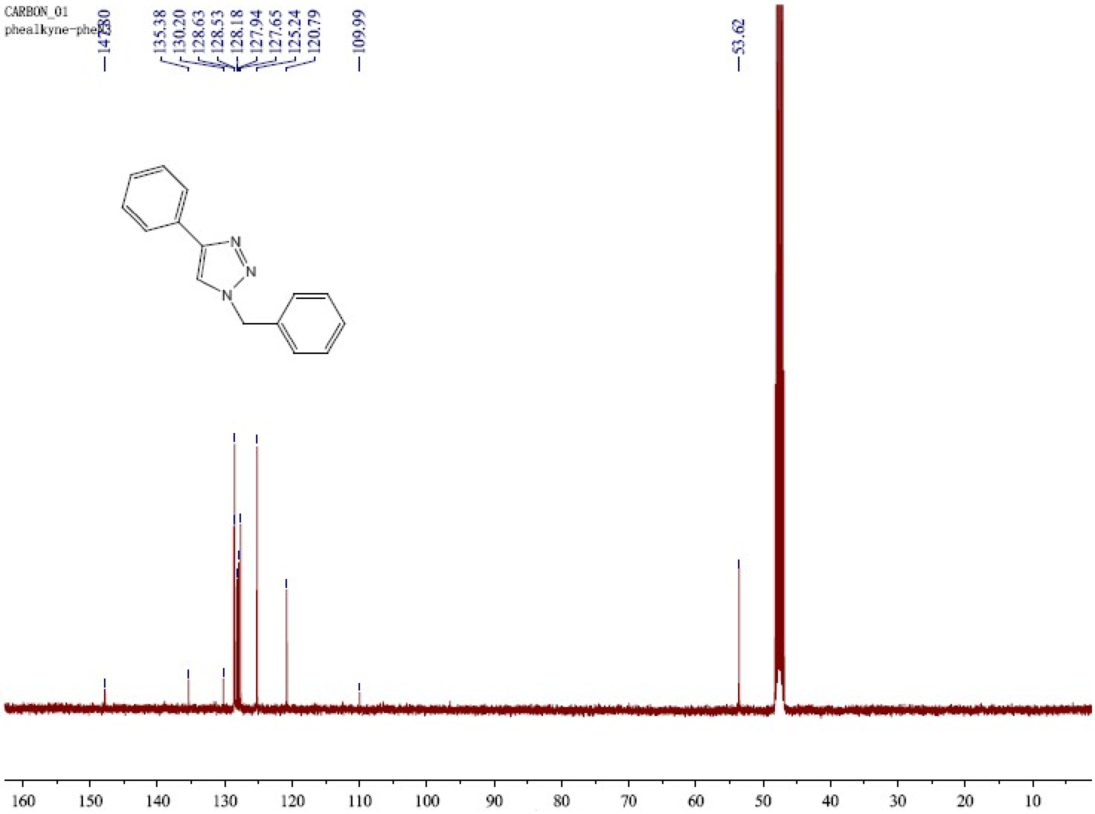

(c). ^{1}H NMR and ^{13}C NMR for 3c

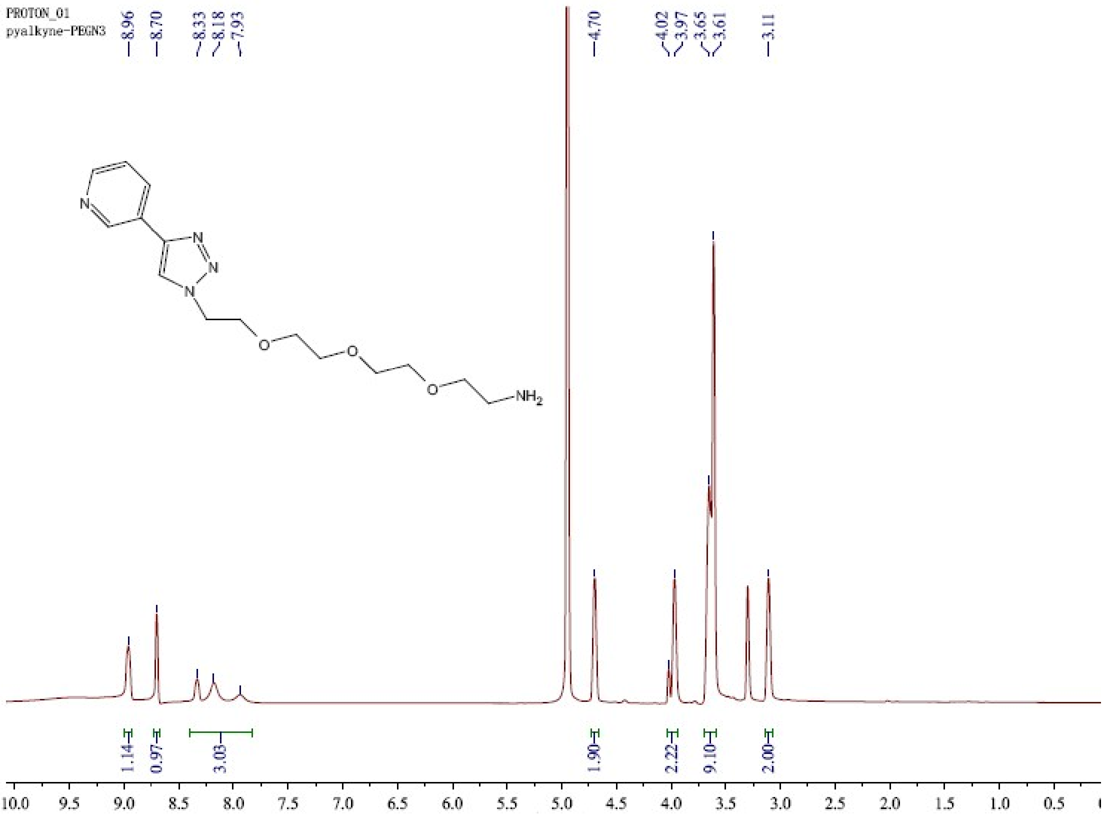

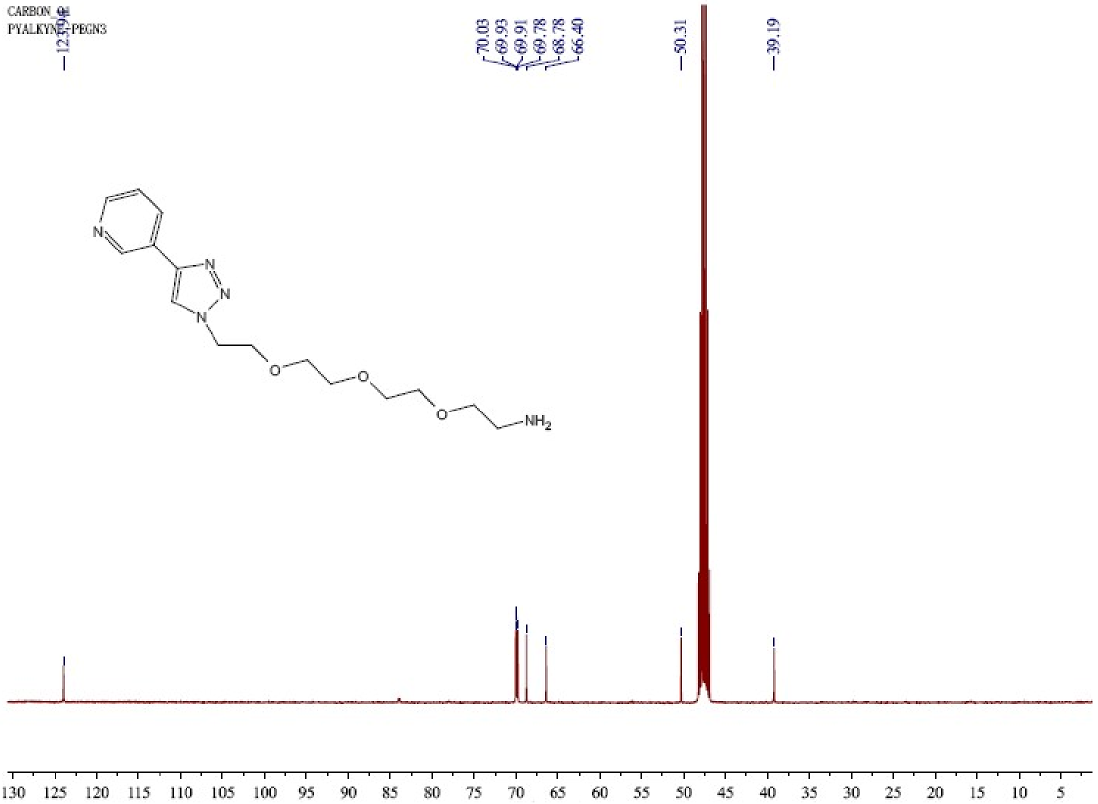

(d). ¹H NMR and for 3d

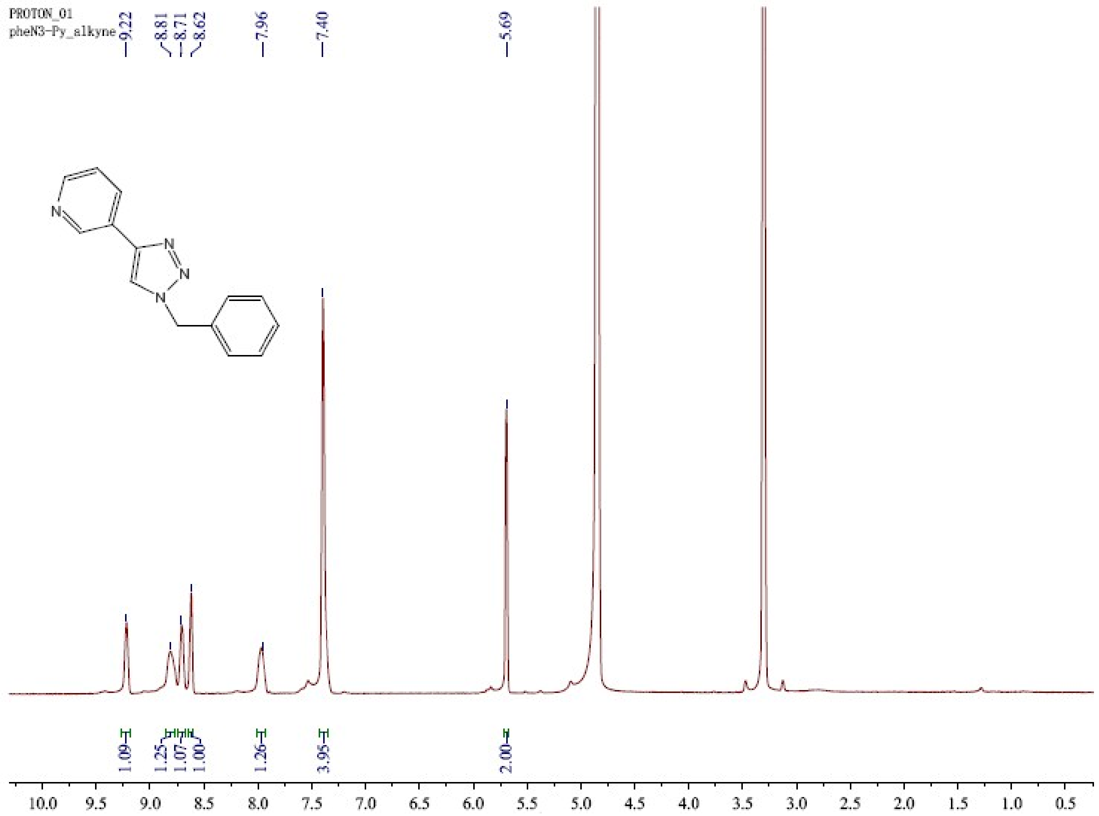

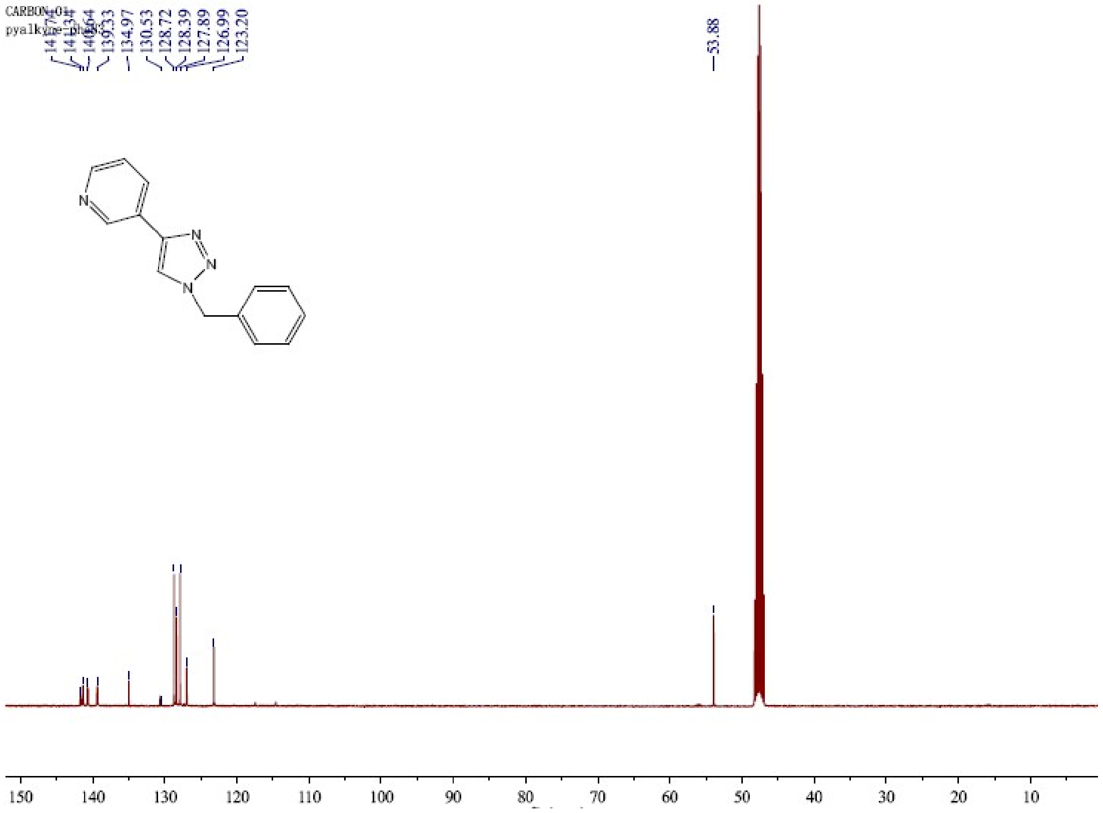

(e). ¹H NMR and for 3e

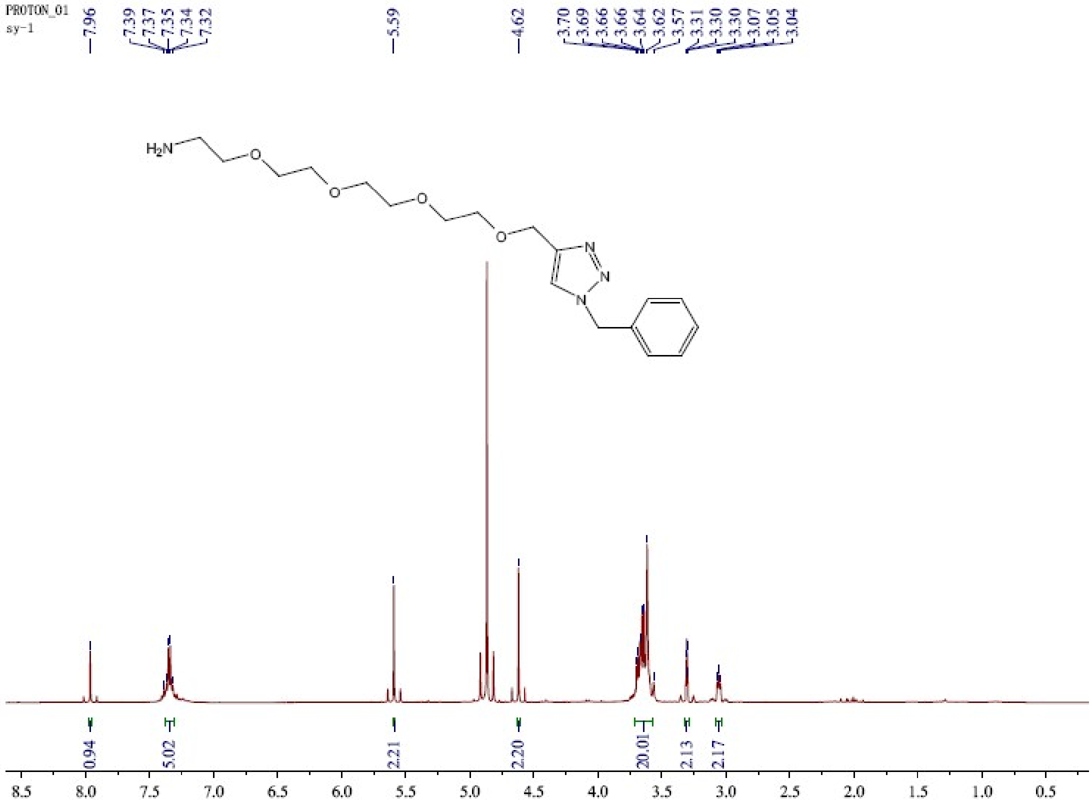

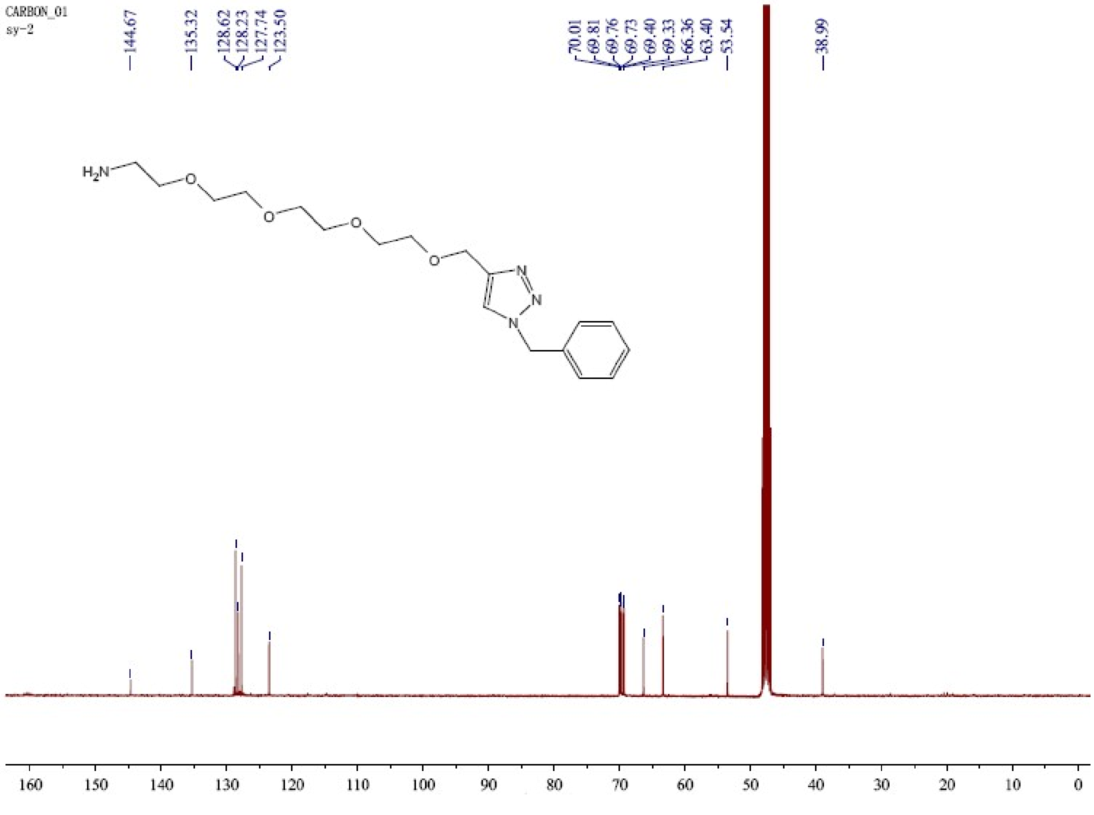

(f). ^{1}H NMR and for 3f

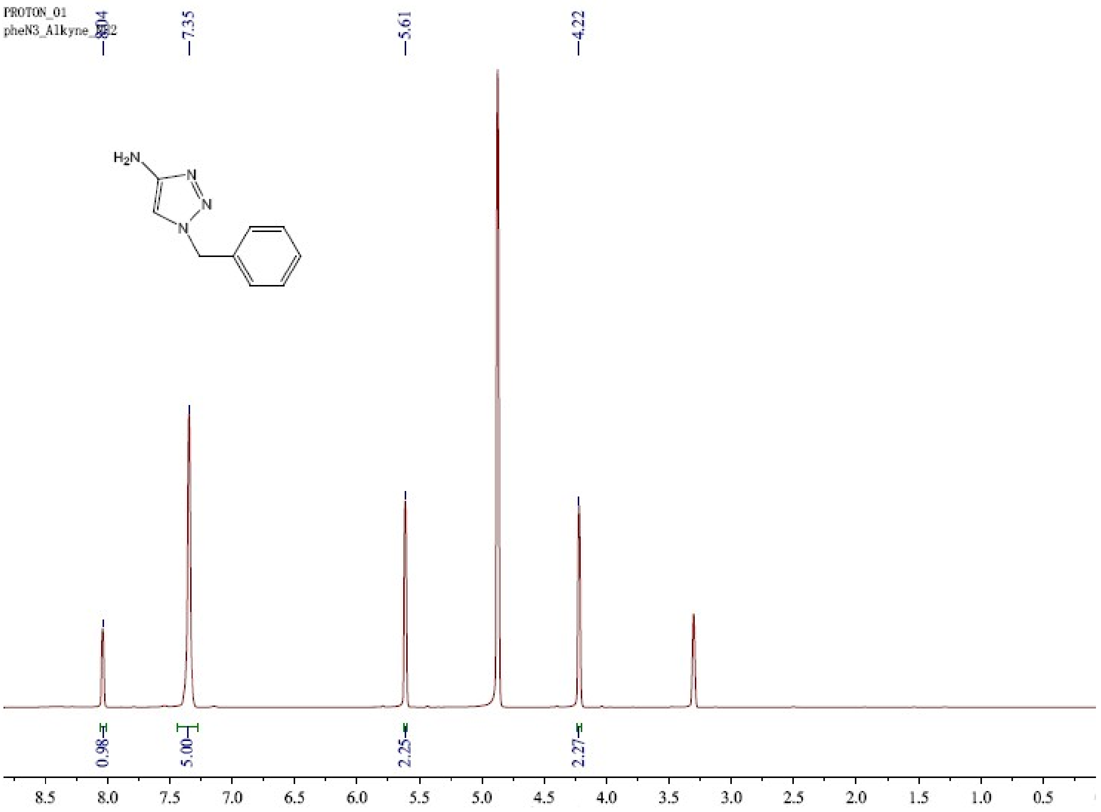

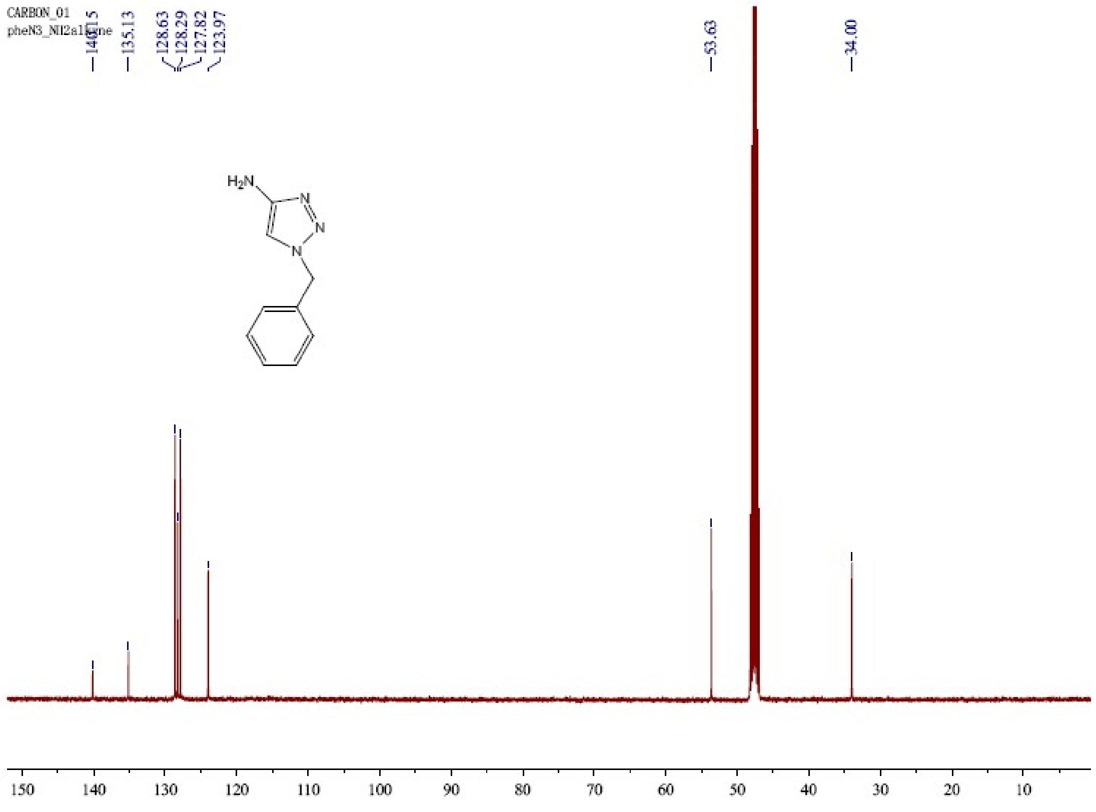

(g). ^{1}H NMR and for 3g

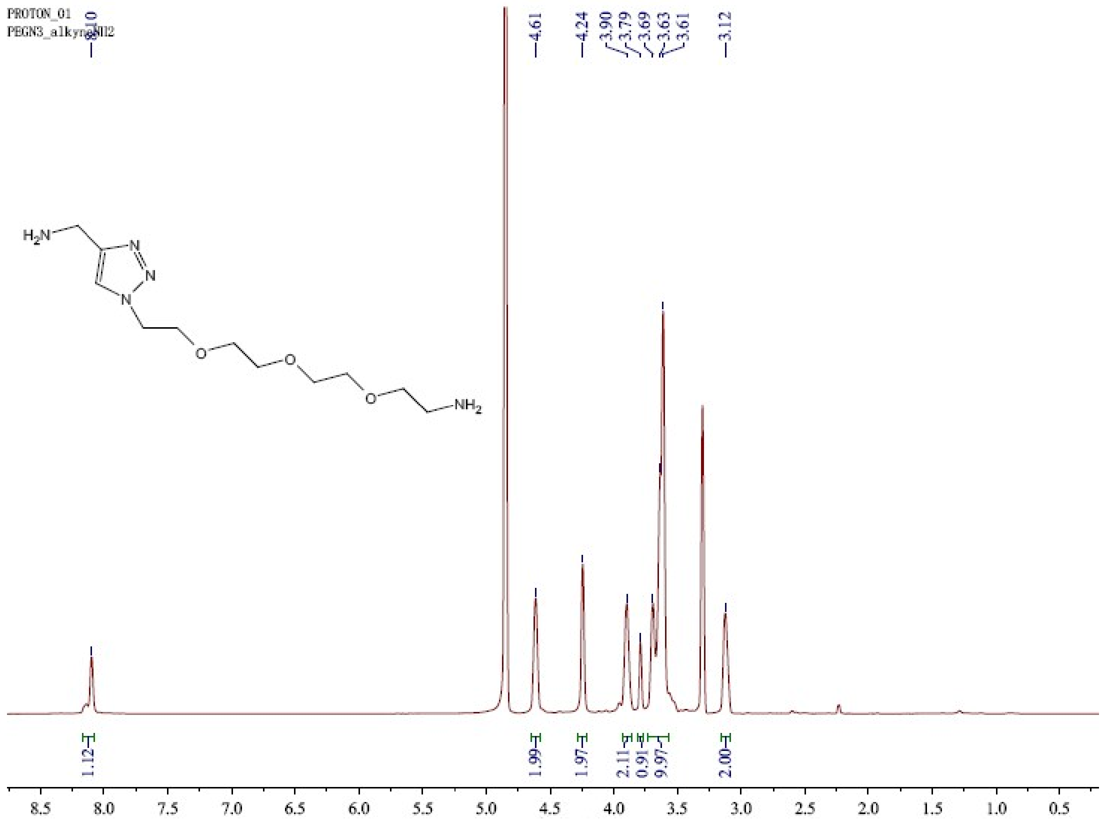

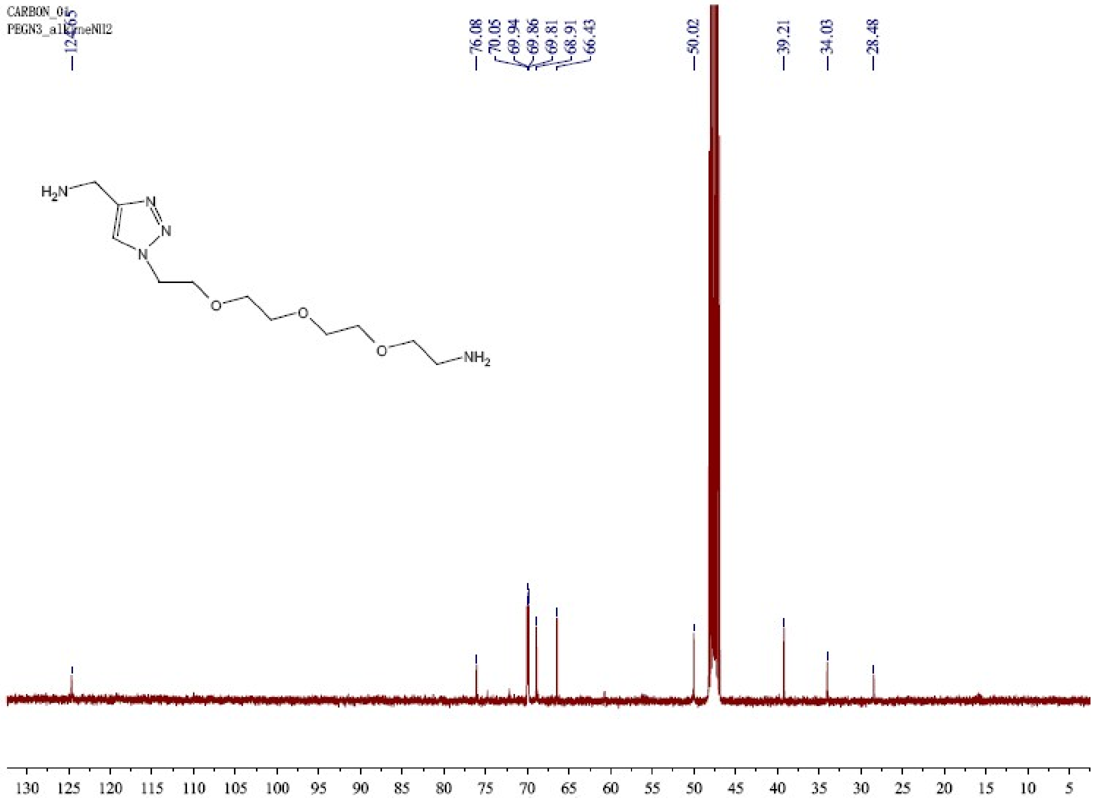

(h). ^{1}H NMR and for 3h

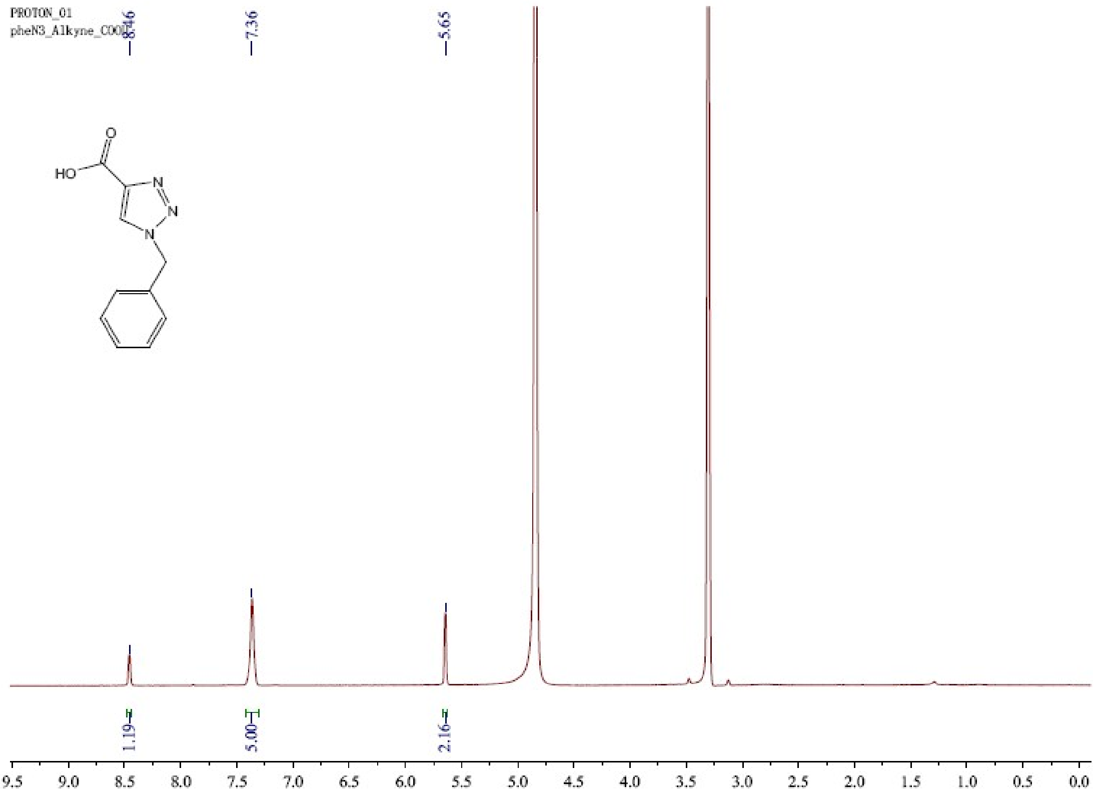

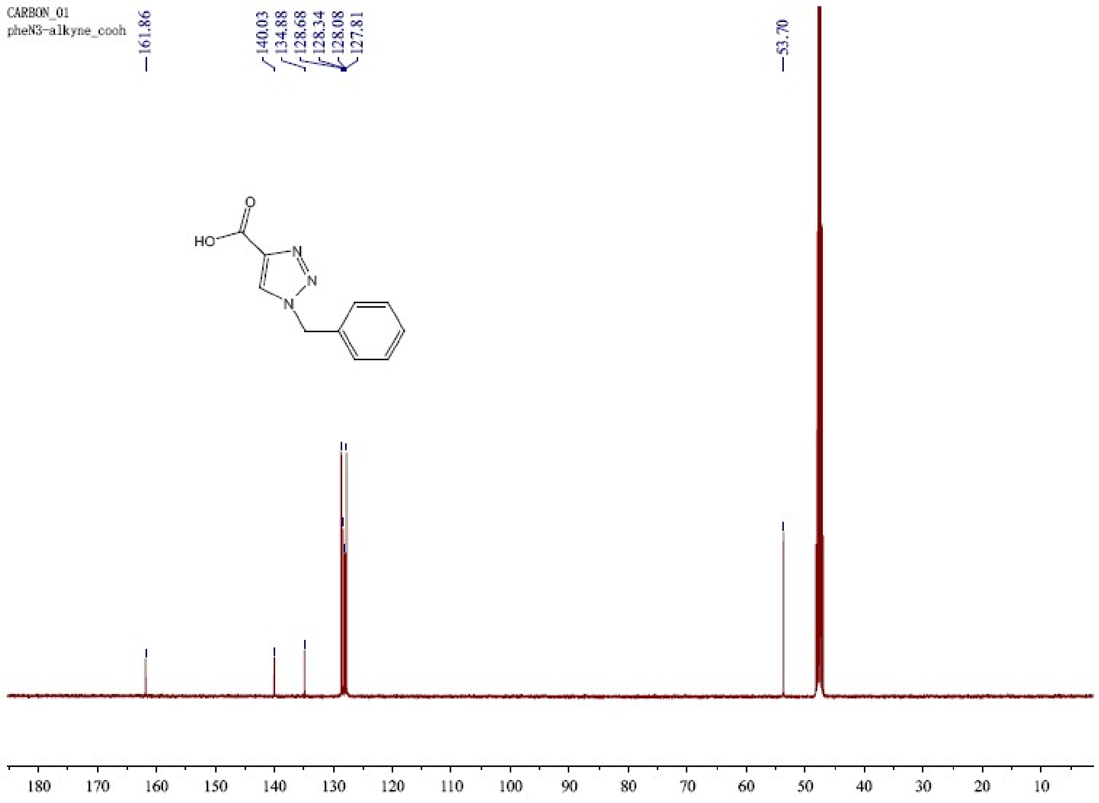

(i). ^{1}H NMR and for 3i

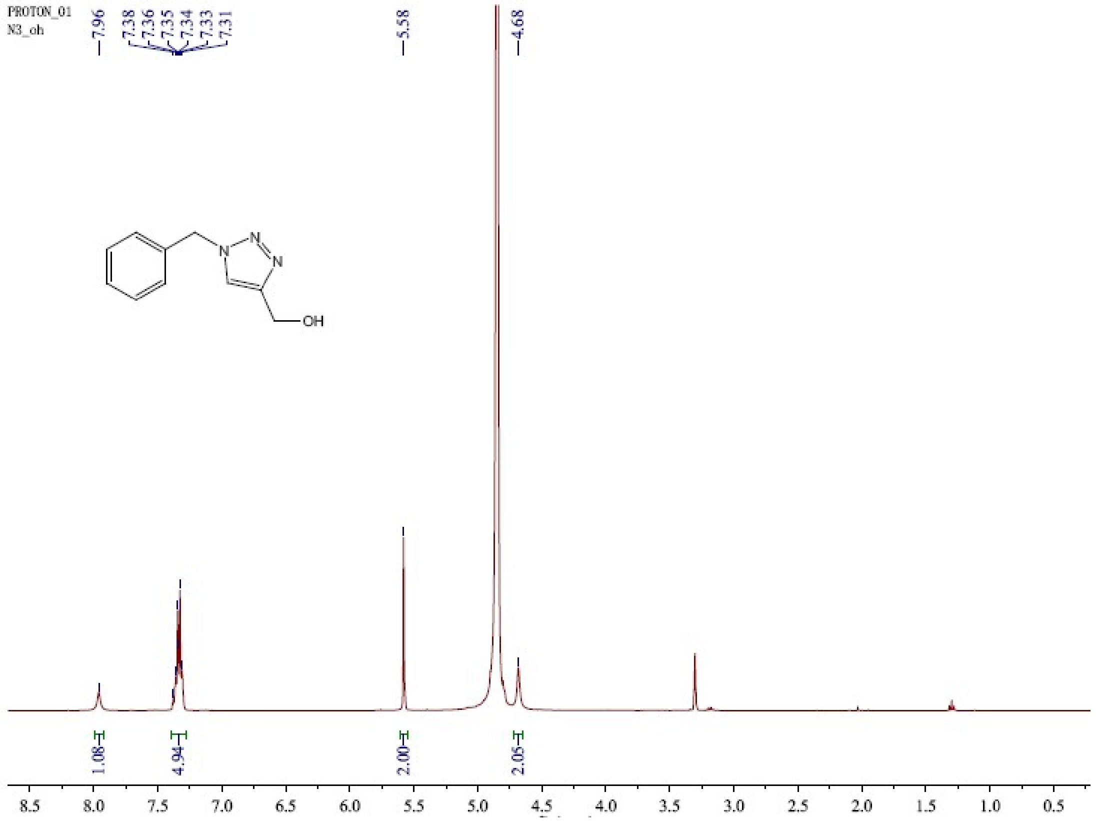

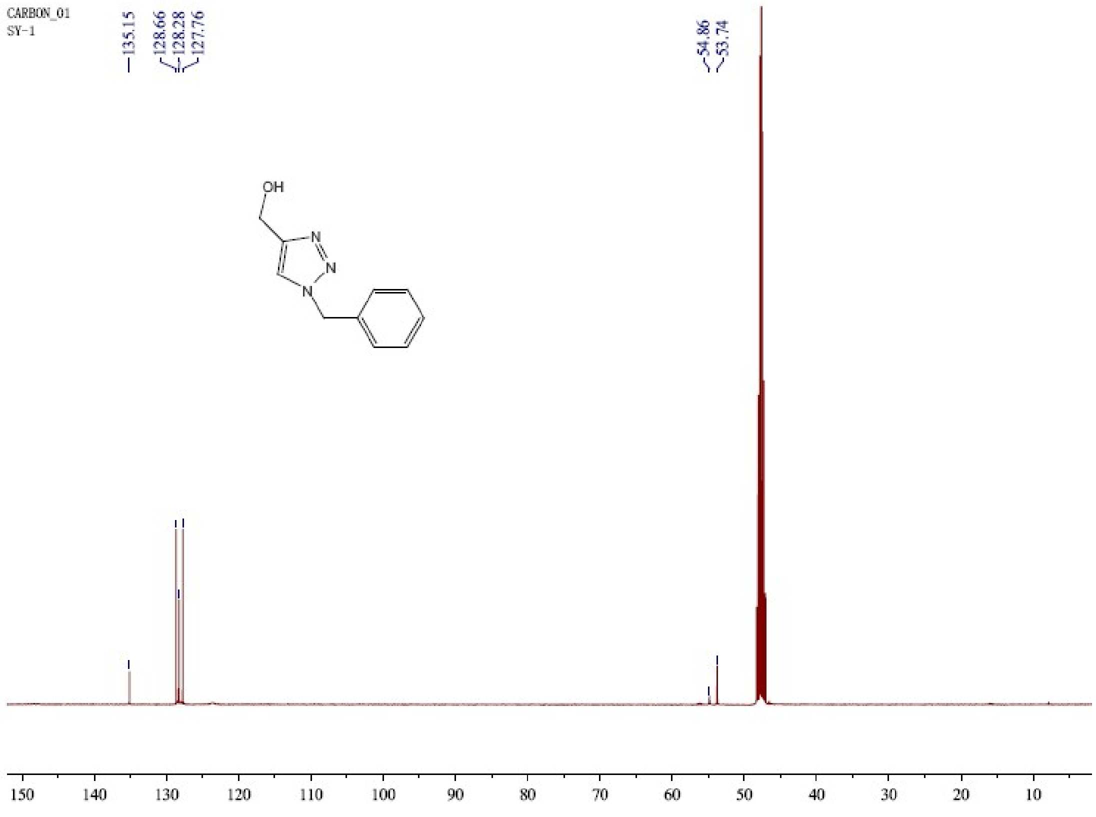

(j). ¹H NMR and for 3j

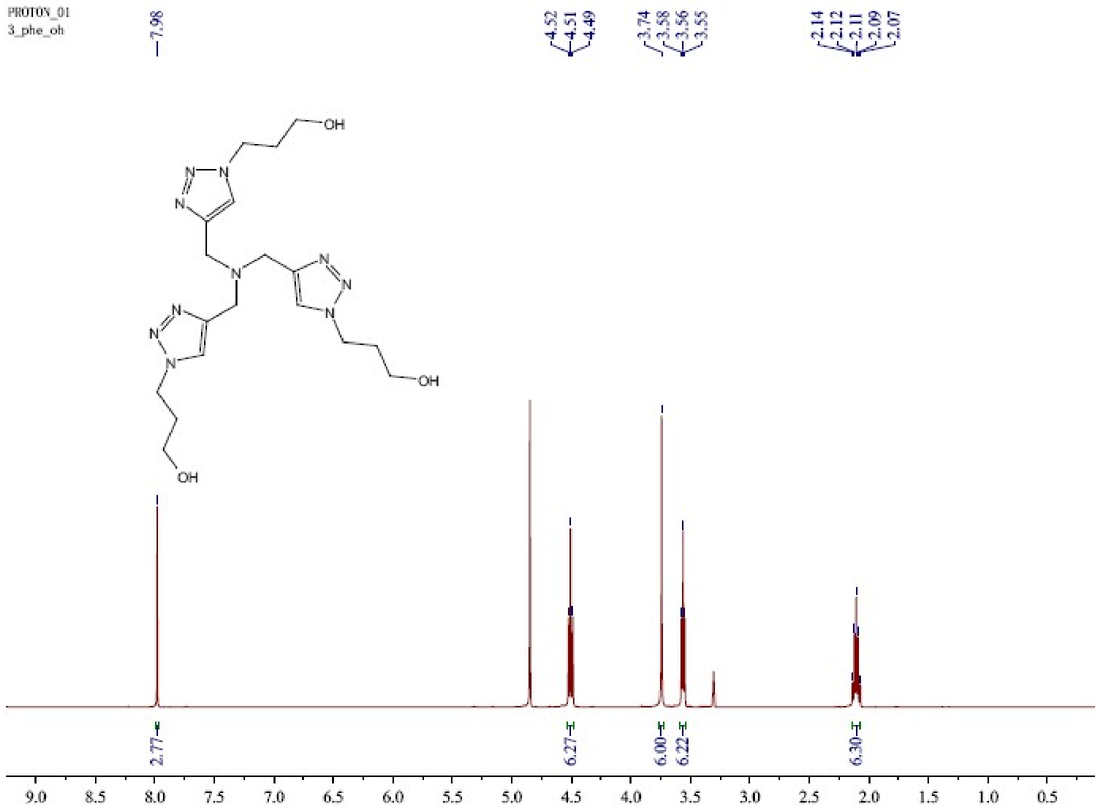

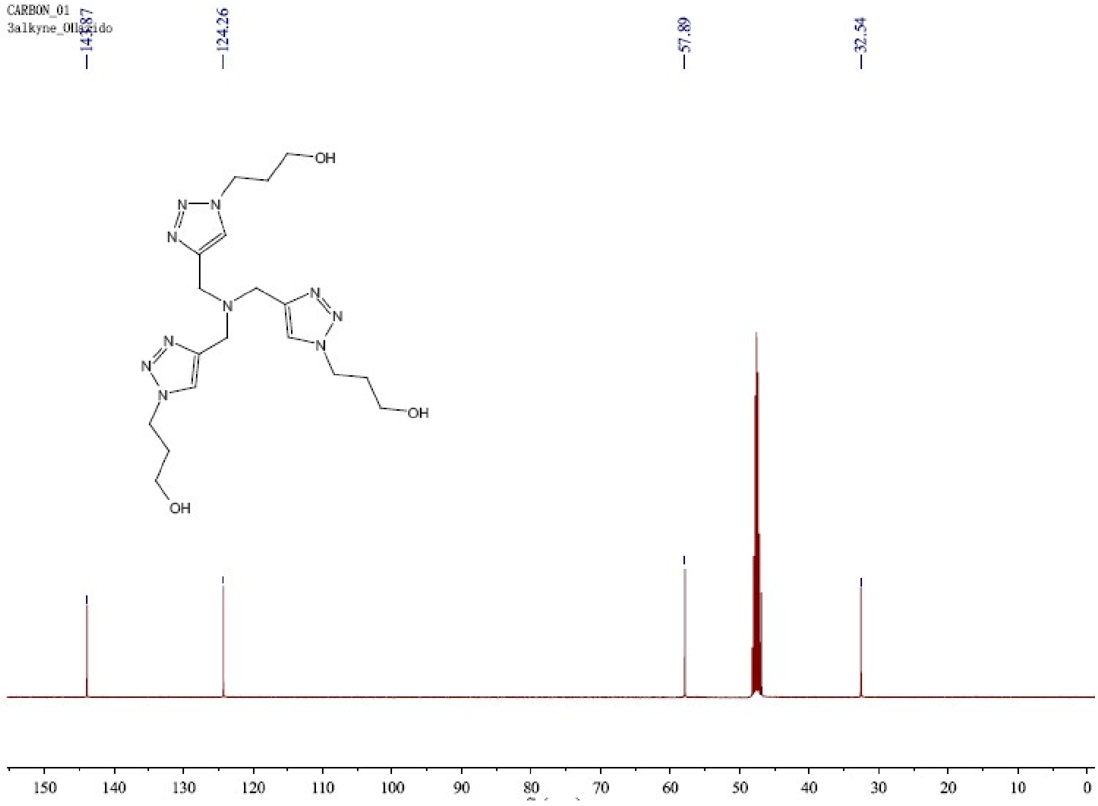

(k). ^{1}H NMR and for 3k

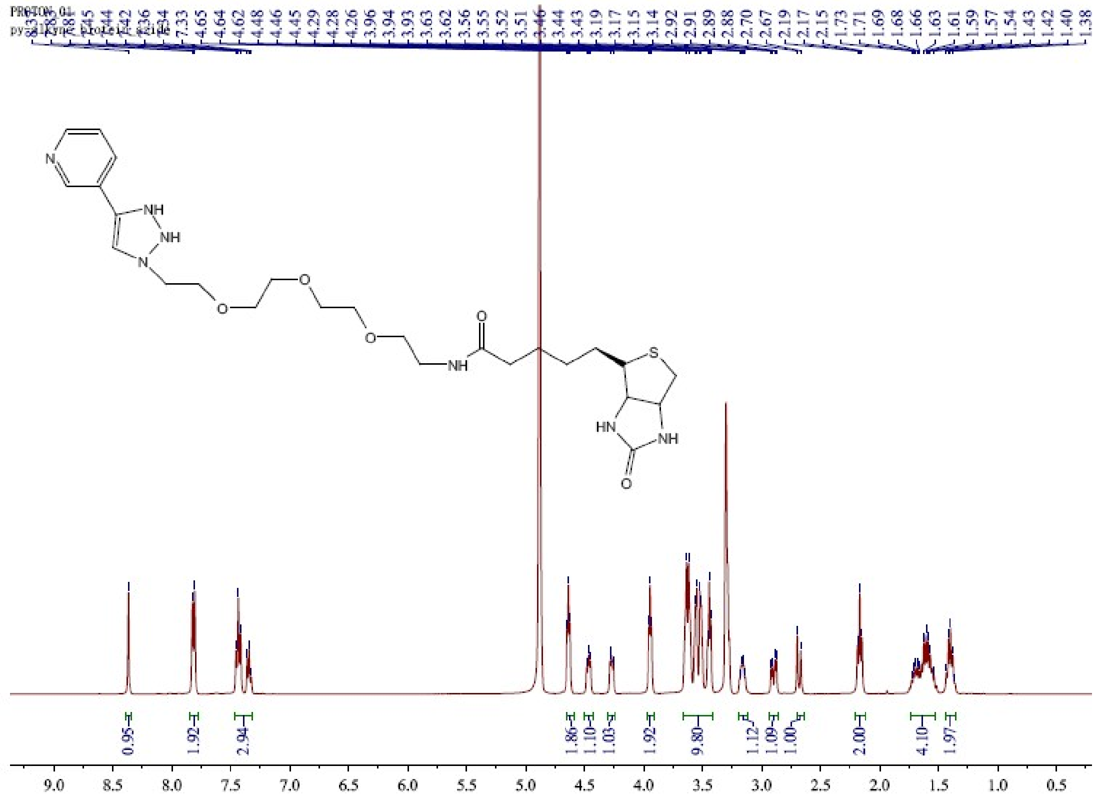

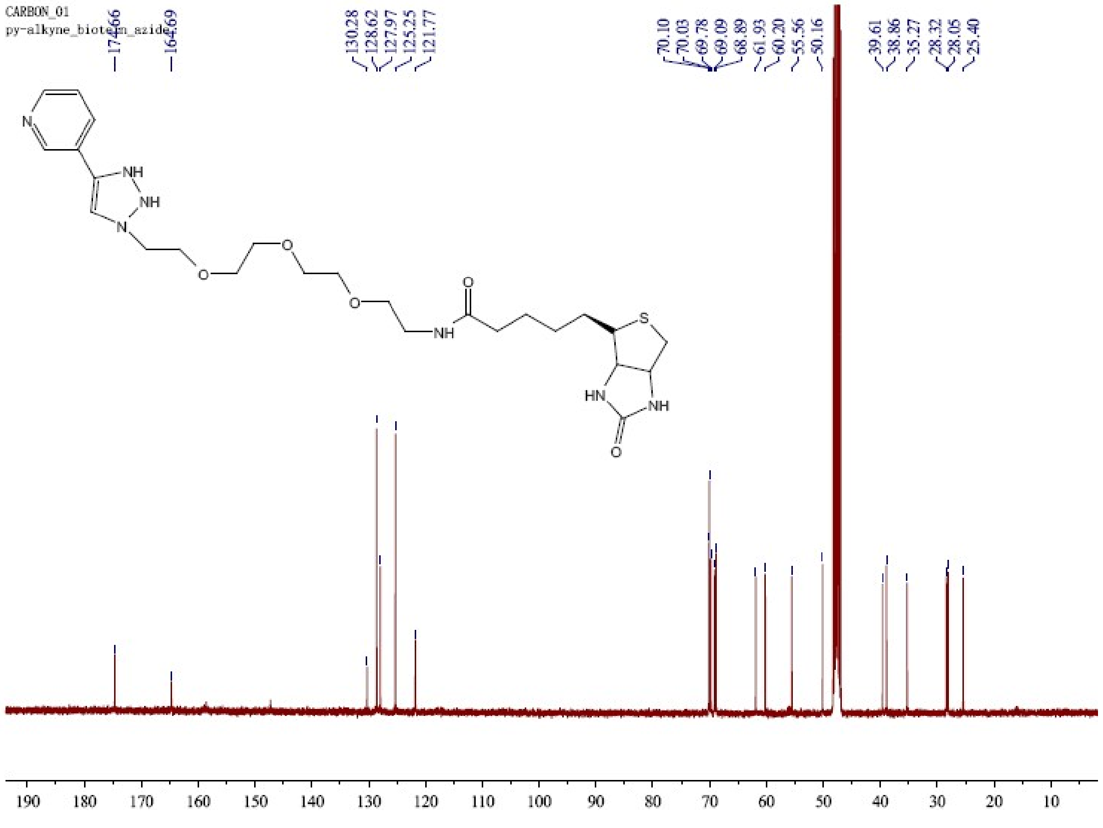

(l). ^{1}H NMR and for 3p

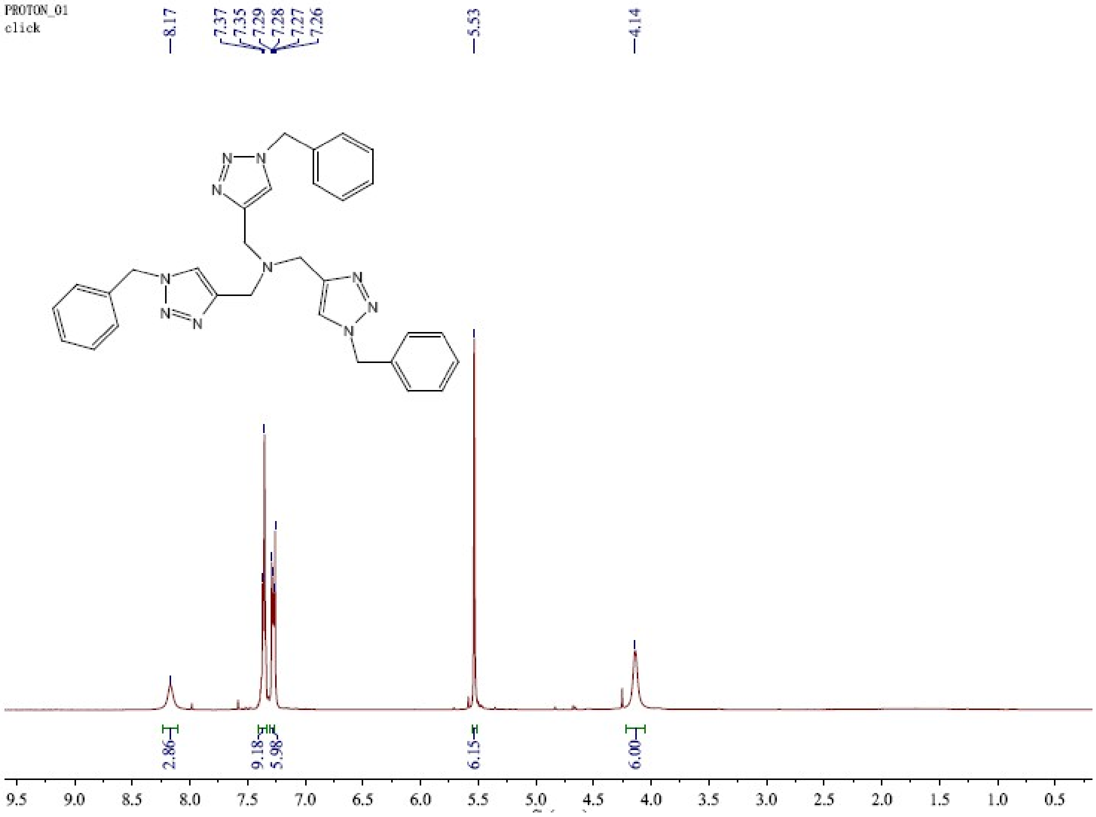

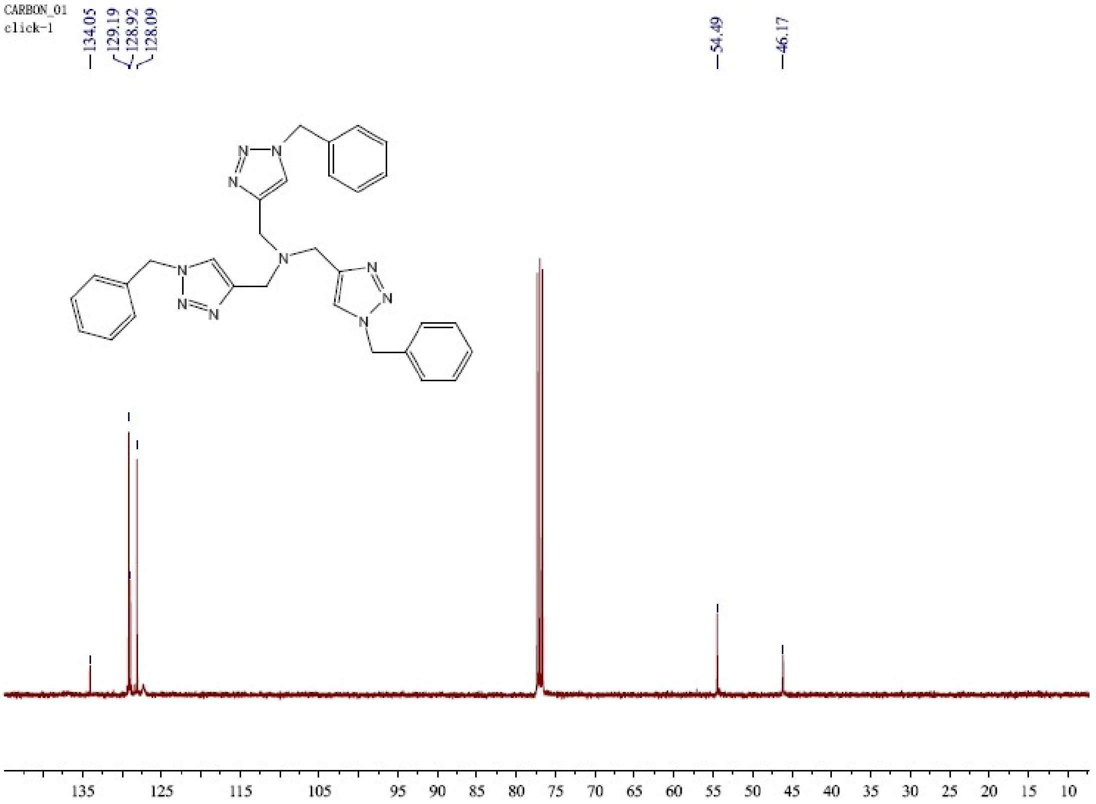

(m). ^{1}H NMR and for 1h

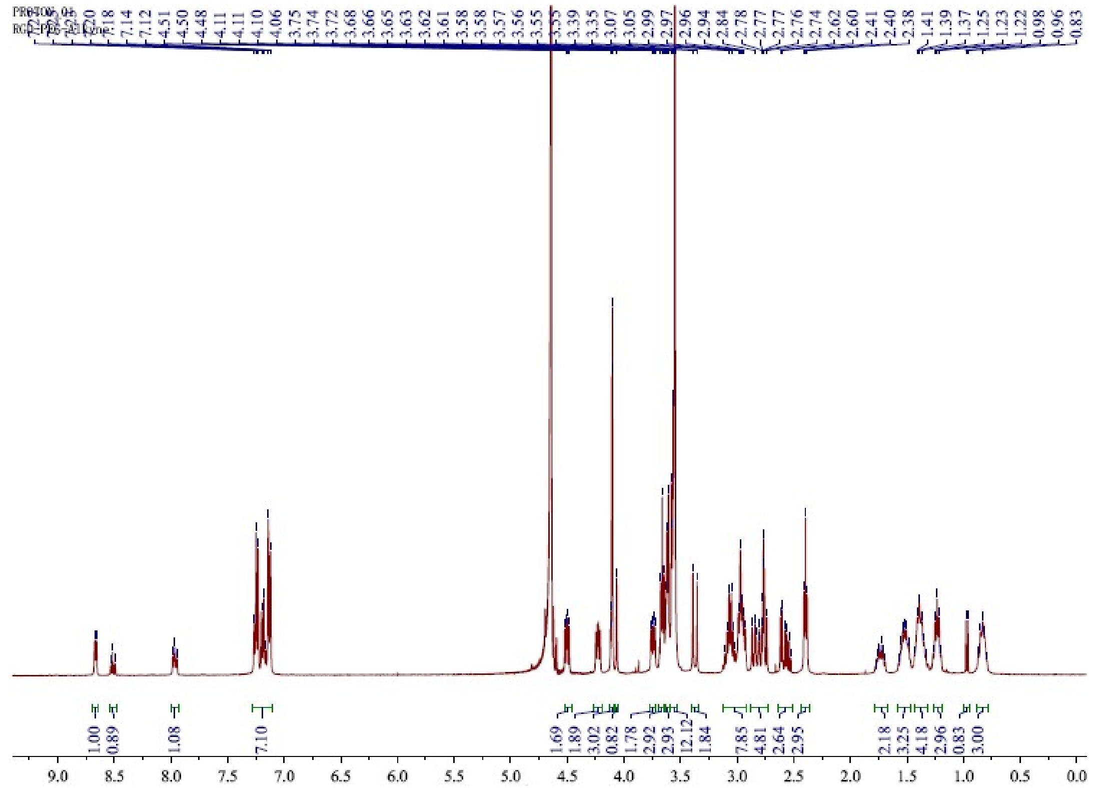

(n). ^{1}H NMR and for 2e

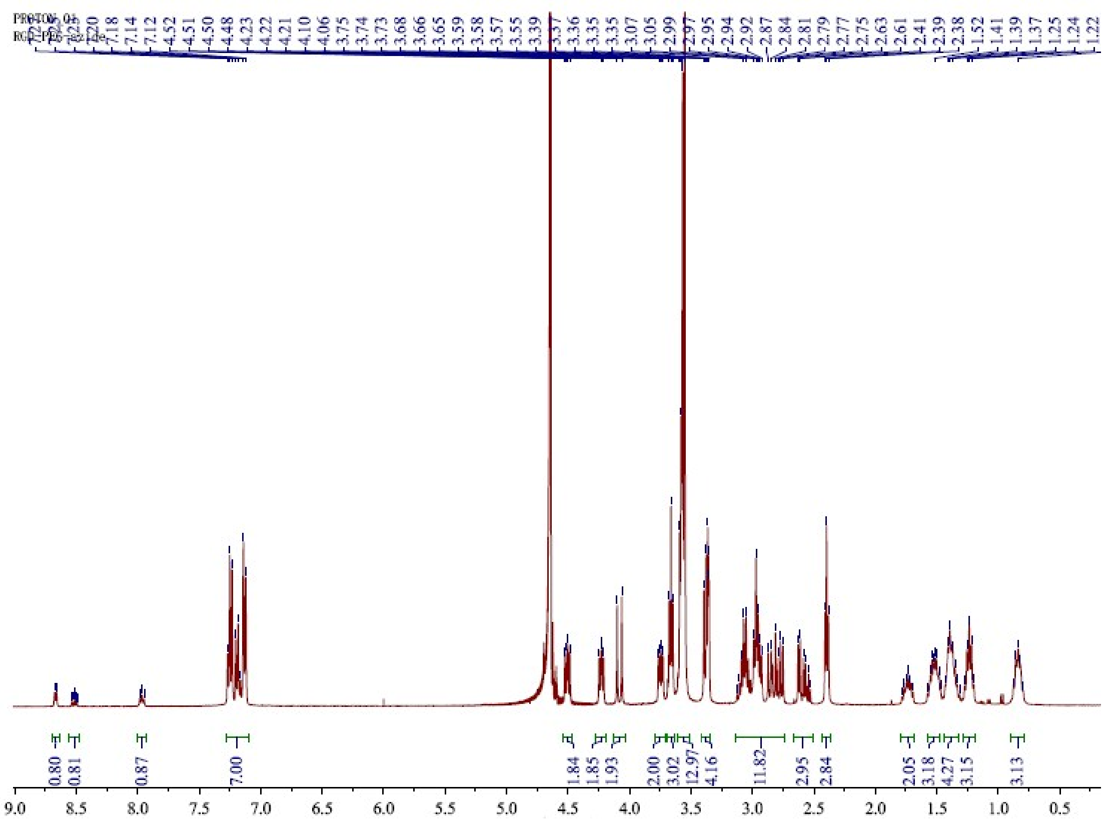

Supplement: Supplementary file 1 [file SC-007-C6SC01536K-s001.pdf]
